# Data, Reagents, Assays and Merits of Proteomics for SARS-CoV-2 Research and Testing

**DOI:** 10.1074/mcp.RA120.002164

**Published:** 2020-11-25

**Authors:** Jana Zecha, Chien-Yun Lee, Florian P. Bayer, Chen Meng, Vincent Grass, Johannes Zerweck, Karsten Schnatbaum, Thomas Michler, Andreas Pichlmair, Christina Ludwig, Bernhard Kuster

**Affiliations:** 1 Chair of Proteomics and Bioanalytics, Technical University of Munich, Freising, Germany; 2 Bavarian Center for Biomolecular Mass Spectrometry (BayBioMS), Technical University of Munich, Freising, Germany; 3 Institute of Virology, School of Medicine, Technical University of Munich, Munich, Germany; 4 German Center for Infection Research (DZIF), Munich partner site, Germany; 5 JPT Peptide Technologies GmbH, Berlin, Germany

**Keywords:** mass spectrometry, label-free quantification, targeted mass spectrometry, parallel reaction monitoring, clinical proteomics, ACE2, COVID-19, SARS-CoV-2, stable-isotope labeling, Vero E6

## Abstract

As the COVID-19 pandemic continues to spread, thousands of scientists around the globe have changed research direction to understand better how the virus works and to find out how it may be tackled. The number of manuscripts on preprint servers is soaring and peer-reviewed publications using MS-based proteomics are beginning to emerge. To facilitate proteomic research on SARS-CoV-2, the virus that causes COVID-19, this report presents deep-scale proteomes (10,000 proteins; >130,000 peptides) of common cell line models, notably Vero E6, Calu-3, Caco-2, and ACE2-A549 that characterize their protein expression profiles including viral entry factors such as ACE2 or TMPRSS2. Using the 9 kDa protein SRP9 and the breast cancer oncogene BRCA1 as examples, we show how the proteome expression data can be used to refine the annotation of protein-coding regions of the African green monkey and the Vero cell line genomes. Monitoring changes of the proteome on viral infection revealed widespread expression changes including transcriptional regulators, protease inhibitors, and proteins involved in innate immunity. Based on a library of 98 stable-isotope labeled synthetic peptides representing 11 SARS-CoV-2 proteins, we developed PRM (parallel reaction monitoring) assays for nano-flow and micro-flow LC–MS/MS. We assessed the merits of these PRM assays using supernatants of virus-infected Vero E6 cells and challenged the assays by analyzing two diagnostic cohorts of 24 (+30) SARS-CoV-2 positive and 28 (+9) negative cases. In light of the results obtained and including recent publications or manuscripts on preprint servers, we critically discuss the merits of MS-based proteomics for SARS-CoV-2 research and testing.

## Teaser

As the COVID-19 pandemic continues to spread, research on SARS-CoV-2 is also rapidly evolving and the number of associated manuscripts on preprint servers is soaring. To facilitate proteomic research, Zecha *et al.* present protein expression profiles of four model cell lines and investigate the response of Vero E6 cells to viral infection. Further, they evaluate the feasibility of proteomic analysis for SARS-CoV-2 diagnostics and critically discuss the merits of proteomic approaches in the context of the COVID-19 research.

## Graphical Abstract

**Figure:**
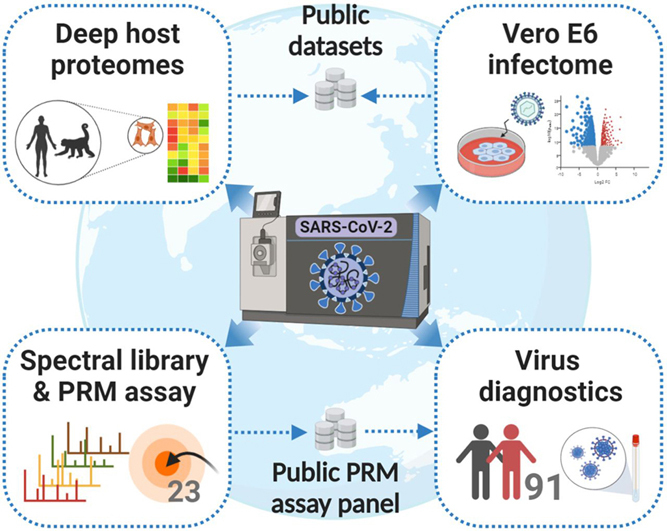

**Highlights**

• In-depth proteomes of 4 SARS-CoV-2 cell line models (Vero E6, Calu-3, Caco-2, A549).
• Proteomic evidence for thousands of *Chlorocebus sabaeus* proteins.
• Proteomic response of Vero E6 cells to SARS-CoV-2 infection.
• Synthetic peptides, spectral libraries, and targeted assays for SARS-CoV-2 proteins.

Mass spectrometry-based proteomics is continuing to make tremendous contributions to life science research and the latest “explosion” of activities around SARS-CoV-2 also motivates proteomic scientists to join efforts that aim at better understanding how this new virus works and how that may inform the development of effective treatments and vaccines. In this context, it is worth reflecting in which areas of the life sciences proteomics has historically been particularly successful. One of the first areas was cell biology. For example, the ability to identify and characterize protein complexes systematically has profoundly changed the way we think about the functional organization of cells. Proteomics then revolutionized the analysis of post-translational modifications to the extent that the vast majority of all PTMs known to date have been found by proteomic approaches. The rapid development of quantitative MS, with or without the use of stable isotopes, paved the way for large-scale cell perturbation studies ranging from growth factors to nutrients to knock-outs or drugs. This has significantly shaped our current understanding of the inner workings of a cell including protein expression regulation, biochemical fluxes and signaling networks to name a few. Today, proteomics is also becoming ever more important in pre-clinical drug discovery and much of the chemical biology field is powered by the ability to interrogate proteins and drugs on a proteome-wide scale. More recently, proteomics has also begun making inroads into structural biology, an area that is undergoing very rapid development and will undoubtedly make important contributions in the future. In comparison, and despite great efforts, progress in clinical proteomics has been slower as many additional challenges present when analyzing complex biology in heterogeneous human populations. That said, recent technological developments and their application suggest that clinical proteomics will become much more successful soon.

In light of the above, it should come as no surprise that proteomics has also become highly successful in virology and a recent special issue of *Molecular and Cellular Proteomics* has highlighted many of the important achievements made in the area of infectious diseases (1). The recent COVID-19 outbreak has spurred a remarkable amount of research activities. At the time of writing, the preprint servers medRxiv and bioRxiv listed ∼5,500 manuscripts for SARS-CoV-2. More than 300 of these mention the term proteomics and a few have already entered the peer-reviewed scientific literature. One example for the latter is a protein interaction map constructed by using affinity-tagged viral proteins and MS that provides an initial overview of how SARS-CoV-2 proteins interact with host proteins (2). Another group applied a pulse-labeling approach to monitor the modulation of the viral translatome and proteome on infection (3), and two laboratories analyzed sera of COVID-19 cases by LC–MS/MS in the search for biomarkers (4, 5). Several studies also present candidate drug targets and small molecules that show antiviral activity *in vitro*. This is of note as controlling the pandemic will require a multitude of measures including effective treatments for patients with severe course of disease using drugs that exist today.

At the beginning of a new research activity, considerable time and effort is required for the molecular characterization of the biological model systems, generating research reagents, and setting up assays. Because the SARS-CoV-2 pandemic puts scientists under heavy time pressure, sharing such resources with the scientific community rapidly can facilitate progress provided that high standards of quality can be upheld. In this report, we contribute high-quality LC–MS/MS data on the proteomes of common cell line models for SARS-CoV-2 research, notably Vero E6, Calu-3, Caco-2, and ACE2-A549 that may be used as a protein expression resource or to build spectral libraries. The African green monkey and derived Vero cell lines often serve as *in vitro* and *in vivo* models for virus research and our analysis exemplifies how mass spectrometric data can be used to improve the annotation of protein-coding regions. Furthermore, we present data on how the virus modulates the proteome of infected cells. In addition, we provide a physical and spectral library of 98 stable isotope-labeled, synthetic peptides representing 11 viral proteins along with optimized PRM assays that were tested on two diagnostic cohorts of in total 91 COVID-19 suspected individuals. Based on our results and examples from the emerging literature, we critically project and discuss the merits of MS-based proteomics for SARS-CoV-2 research and testing.

## EXPERIMENTAL PROCEDURES

##### Experimental Design and Statistical Rationale

The rationale of the experimental design is described in more detail in the respective method and result sections and in the supplemental Methods. In brief, we first aimed to characterize the protein expression profiles of three model cell lines (African green monkey Vero E6 kidney cell line, human Caco-2 colon and Calu-3 lung-cancer cell lines) commonly used in virology studies. In addition, the human A459 lung cancer cell line stably transfected with ACE2, a peptidase reported to serve as entry point for SARS-CoV-2 into cells (6) was included for deep proteome profiling. To this end, we performed deep proteome analyses measuring 48 basic reversed phase (RP) fractions for each cell line and generating high resolution and mass accuracy fragment spectra. For Vero E6 and ACE2-A459 cells, a workflow replicate was prepared by employing a faster, but lower resolution method for MS2 spectra acquisition. High resolution and mass accuracy MS2 spectra from Vero E6 cells and a database search including human protein sequences were used further to exemplify a proteomics-guided refinement of the expressed genome and identify genes or parts of genes that have been completely missed in the African green monkey genome annotation provided by Uniprot and/or RefSeq. Next, the response of Vero E6 cells 24 h after SARS-CoV-2 infection at 2 different multiplicities of infection (MOI) was investigated in cell culture triplicates to enable analyses of significant protein expression changes. In addition, obtained infectome data were compared with a recently published virus-host response study (3) and a SARS-CoV-2 interactome study (2). Finally, using heavy synthetic peptide references, we generated a spectral library entailing fragment ion spectra and retention time information for 98 SARS-CoV-2 peptides. This was refined further to a PRM assay panel containing 23 peptides and applied to the detection of SARS-CoV-2 in two clinical cohorts. In total, 91 respiratory specimens, of which 37 were tested negative and 54 were tested positive for SARS-CoV-2 by RT-PCR (RT-PCR), were analyzed by nano- and micro-flow PRM using two different input quantities. All significance and enrichment analyses were corrected for multiple testing at 5% FDR. Further, instead of choosing a p-value cut-off, S0 was specified to adjust the significance cut-off of statistical analyses on the fold-change level in a data-driven way while accounting for differing variances across the range of measured values and groups. For two-sided t-tests, at least 2 valid quantifications per group were required, and equal variances were assumed for each group as well as normal distribution of log transformed protein intensities. To characterize correlations, Pearson correlation coefficients (R) were computed under the assumption of a linear relationship between two variables.

##### Synthetic Peptides and Antibodies

Isotopically labeled SpikeTides^TM^ peptides covering 11 SARS-CoV-2 proteins were kindly provided by JPT Peptide Technologies (for details see supplemental Methods). All quantities per spike-in peptide specified in the following represent only rough estimates, as the isotopically labeled peptides were not purified and concentrations were not determined accurately. For retention time calibration, PROCAL retention time peptides from JPT Peptide Technologies (7) and indexed retention time (iRT) peptides from Biognosys (8) were used. Western blots were performed according to standard procedures using 30 µg protein as input and antibodies against human ACE2 (R&D Systems, Cat# AF933, 1 µg/ml) and β-actin (Santa Cruz Biotechnology, sc-47778, 1:500).

##### Cell Treatment and Lysis

Details of cell culture conditions, the generation of a cell line expressing ACE2, and virus growth and virus titer and cell viability assays are specified in supplemental Methods. For investigation of the host cell response to the virus, 10e6 Vero E6 cells were infected with mock or SARS-CoV-2-MUN-IMB-1 strain at a MOI of 3 or 0.1, and triplicates of each condition were lysed 24 h post infection. Supernatant of infected Vero E6 cells was collected 48 h post infection using a MOI of 0.01 and spun twice at 1000 × *g* for 10 min All cells were lysed in SDS lysis buffer (2% SDS in 40 or 50 mm Tris/HCl pH 7.6), and virus-containing cell lysates and supernatant were heated at 95°C for 5 to 10 min before storage at −80°C.

##### SP3 Protein Digestion, TMT Labeling and Fractionation of Cell Line Samples

To hydrolyze DNA and reduce viscosity, cell lysates were heated at 95°C for 5 min and TFA was added to a final concentration of 1% (9). Quenching was performed using 3 M Tris, pH 10 (final concentration of ∼195 mm, pH 7.8). Protein concentration was determined using the Pierce BCA Protein Assay Kit (Thermo Scientific). Proteins were cleaned up and digested using the SP3 method on an automated Bravo liquid handling system (Agilent) as previously described (10) with minor modifications, details of which are specified in the supplemental Methods. In brief, 1 mg of a 1:1 mix of two types of carboxylate beads (cat# 45152105050250 and 65152105050250, GE Healthcare), 200 µg of protein digest (120 µg for Vero E6 measured with ion trap MS2 method) and a 1:50 trypsin-to-protein ratio for overnight digestion at 37 °C were used. Peptides were desalted using RP-S cartridges (5 μl bed volume, Agilent) and the standard peptide cleanup v2.0 protocol on the AssayMAP Bravo Platform (Agilent, wash solvent: 0.1% FA; elution solvent: 0.1% FA in 70% ACN). Triplicates of SARS-CoV-2 infected Vero E6 cells (30 µg of peptides per replicate) were labeled with 9 channels of TMT10plex reagent kit (Thermo Scientific, channel 127N was omitted) according to our previously published protocol (11) with minor modifications as specified in the supplemental Methods. After vacuum drying, TMT-labeled peptides were dissolved in 0.1% FA and desalted by the AssayMAP Bravo Platform (Agilent) as described above. For off-line high pH reversed phase (RP) fractionation of label-free and TMT-labelled cell lines, a Dionex Ultra 3000 HPLC system equipped with a Waters XBridge BEH130 C18 column (3.5 μm 2.1 × 150 mm) was operated at a flow rate of 200 µl/min with a constant 10% of 25 mm ammonium bicarbonate (pH = 8.0) in the running solvents. Nonlabeled peptides (200 µg) were separated using a 57 min linear gradient from 4 to 32% ACN in ddH2O followed by a 3 min linear gradient up to 85% ACN. For TMT-labeled peptides, a 57 min linear gradient from 7 to 45% ACN in ddH2O followed by a 6 min linear gradient up to 80% ACN was employed. Forty-eight fractions were collected every half minute from minute 3 to 51 and pooled discontinuously into 48 fractions (fraction 1 + 49, fraction 2 + 50, and so on). Peptide fractions were frozen at −80°C and dried by vacuum centrifugation.

##### Sample Preparation of a Supernatant from SARS-CoV-2 Infected Vero E6 Cells

Supernatant of SARS-CoV-2 infected Vero E6 cells, which contained 2e6 virions (infectious virus particles) per ml as measured by plaque assay, was used to evaluate SP3-based, in-gel and in-solution digestion in urea buffer for the detection of SARS-CoV-2 derived peptides (see supplemental Methods for details). Further, a dilution series was prepared from the virus supernatant sample in 8 steps (15, 5, 1.5, 0.5, 0.15, 0.05, 0.015, and 0.005 μg of total protein amount). Dilutions were used as input for the in-gel digestion workflow by mixing them 1:1 with 4× Novex NuPAGE LDS sample buffer (Invitrogen) containing 20 mm DTT. Samples were run 1 cm into a 4–12% Bis-Tris-protein gel using 1× MOPS SDS running buffer (Novex NuPAGE, Invitrogen). Reduction, alkylation, and overnight digestion of proteins (using 250 ng trypsin) were performed according to standard in-gel procedures. In parallel, gel bands loaded with sample buffer only were processed representing “blank” samples. The identical amount (∼15 fmol) of isotopically labeled SARS-CoV-2 peptide mix was added to all 9 samples. Subsequently, one-third of the sample was measured by nano-flow and two-third by micro-flow PRM targeting 23 and 21 SARS-CoV-2 peptides, respectively.

##### Sample Preparation of Respiratory Specimens

Details on the collection of respiratory specimens and the determination of their virus load in genome equivalents (geq) via RT-PCR are given in the supplemental Methods. In this study, 91 specimens that were collected as part of the standard diagnostic testing and would normally be discarded were used. Approval to do so was granted by the ethics committee of the University Hospital “rechts der Isar” of the Technical University of Munich. Person identification was not recorded, and only SARS-CoV-2 proteins were investigated. For nano-flow PRM analysis of clinical cohort 1, 15 µl of residual material from testing of 52 diagnostic samples was mixed 3:1 with 4× Novex NuPAGE LDS sample buffer containing 40 mm DTT and used as input for in-gel digestion using 250 ng trypsin. Isotopically labeled SARS-CoV-2 peptide mix (∼5 fmol/injection), PROCAL retention time peptides and iRT peptides were spiked into all 52 clinical samples directly before measurement. For the micro-flow PRM measurements of cohort 1, in total 50 µl of each sample were mixed with 4× LDS sample buffer containing 40 mm DTT, added to two gel pockets, and combined after digestion using 500 ng trypsin per gel lane. For cohort 2, up to 300 µl of 39 nasopharyngeal swab samples were dried down and resuspended in 25 µl of 2× Novex NuPAGE LDS sample buffer containing 10 mm DTT before subjection to in-gel digestion using 1 µg of trypsin. Before micro-flow PRM measurement, heavy SARS-CoV-2 peptides (∼50 fmol/injection), PROCAL, and iRT peptides were spiked into all samples. For the nano-flow setup, all peptides corresponding to 5 µl of the original sample were used, whereas for micro-flow analyses a quantity corresponding to 46.4 µl of the original sample was injected into the MS (equivalent to the input amount for standard RT-PCR diagnostic analysis). As negative/blank controls, empty gel lanes were processed and analyzed in parallel with all clinical samples.

##### Setup of PRM Assays for ACE2, TMPRSS2, and SARS-CoV-2 Proteins

All PRM assays were designed in accordance with the Tier 3 guidelines for targeted assay development (12, 13). In addition, isotopically labeled reference peptides were used for detection of SARS-CoV-2 derived peptides to achieve maximally confident identification. A more detailed description of different PRM assays can be found in the supplemental Methods section. In brief, peptides for ACE2 and TMPRSS2 were selected based on most intense peptides in data dependent acquisition (DDA) measurements of high pH RP fractions. For monkey proteins, peptides that are identical or correspond to a human peptide that has been identified in any of the human cell lines were additionally included. In total, 15/6/9/6 peptide sequences were targeted for human ACE2/TMPRSS2/monkey ACE2/TMPRSS2 proteins. Spectral libraries were built from experimental spectra of deep proteome measurements and predicted spectra using Skyline (version 20.1.1.83) (14) and the Prosit 2019 algorithm (15) and are available for download from Panorama Public (16) (https://panoramaweb.org/SARS-CoV-2.url). For peptides that have not been identified in DDA runs, retention time was predicted using Prosit.

SARS-CoV-2 peptide selection started with the *in silico* tryptic digestion of the Uniprot derived SARS-CoV-2 proteome (UP000464024, 14 entries, last modified on 22nd of March 2020). In total, 113 peptides representing 11 proteins met our selection criteria. All peptides were synthesized as SpikeTides^TM^ in isotopically labeled form (JPT Peptide Technologies) and pooled into a single peptide mix. Spectral libraries of 98 confidently detected peptides (MaxQuant score > 50) containing high-quality reference spectra and retention time information were built from experimental spectra of synthetic peptides and predicted spectra using Skyline and the Prosit algorithm and are available for download from Panorama Public (16) (https://panoramaweb.org/SARS-CoV-2.url). The assay panel was further refined for the detection of SARS-CoV-2 in respiratory specimens using supernatant sample and based on uniqueness for SARS-CoV-2 and the highest endogenous PRM-MS2 signal using the top 6 fragment ions from the spectral library. Finally, we derived a panel of 23/21 optimal PRM assays for SARS-CoV-2 detection using nano-/micro-flow PRM and a 50/15-min linear gradient.

### LC–MS/MS Measurements

##### Label-Free Cell Line Proteomes

For LC-ESI-MS/MS measurement of deep-scale proteomes, a Dionex UltiMate 3000 RSLCnano System equipped with a Vanquish pump module and coupled to a Fusion Lumos Tribrid mass spectrometer (Thermo Fisher Scientific) was operated under micro-flow conditions as we described recently (17). Peptide fractions were dissolved in 1% FA containing 500 fmol of PROCAL peptides per injection, and the total fraction corresponding to ∼3.75 to 5 µg peptides was injected directly onto a commercially available Acclaim PepMap 100 C18 LC column (2 μm particle size, 1 mm ID × 150 mm; Thermo Fisher Scientific). Peptides were separated at a flow rate of 50 µl/min using a 15 min linear gradient of 3 to 28% micro-flow solvent B (0.1% FA and 3% DMSO in ACN) in micro-flow solvent A. The Fusion Lumos was operated in DDA and positive ionization mode using an H-ESI source. All four cell lines were measured employing a high-resolution orbitrap (OT) method to obtain high-quality MS2 scans. In brief, full scan MS1 spectra were recorded in the OT from 360 to 1,300 m/z at 60 k resolution using an automatic gain control (AGC) target value of 4e5 charges and a maximum injection time (maxIT) of 50 ms. The RF-lens level was set to 40%. MS2 spectra were acquired in the OT at 15 k resolution after higher energy collisional dissociation (HCD, 32% normalized collision energy (NCE)) and using an AGC target value of 1e5 charges, a maxIT of 25 ms, an isolation window of 1.2 *m*/*z*, and an intensity threshold of 2.5e5. The ‘inject beyond’ functionality was enabled to use available parallelizable time. The cycle time was set to 0.6 s and the dynamic exclusion lasted for 12 s. Workflow replicates of Vero E6 and ACE2-A549 cells were additionally analyzed with a faster, but lower resolution ion trap (IT) method with the following modifications compared with the OT method: Full scan MS1 spectra were recorded in the OT at 120 k resolution. MS2 spectra were acquired in the IT using the rapid scan mode after precursor isolation using an isolation window of 0.4 *m*/*z*, an AGC target value of 1e4, a maxIT of 10 ms, and an intensity threshold of 5e4 charges.

##### TMT-Labeled Infectome

For MS3-based measurements of TMT-labeled Vero E6 peptides, the following parameters were adjusted compared with the deep-scale proteomic analysis with IT readout as described above: A 25 min linear gradient from 4 to 32% micro-flow solvent B in A was applied. The scan range was increased from 360 to 1560 *m*/*z* and the RF-lens level to 50%. IT-MS2 spectra for peptide identification were acquired after collisional dissociation (CID) by resonance activation in the IT for 10 ms with a q-value of 25 and using an isolation window of 0.6 *m*/*z*. To obtain quantitative information on TMT reporter ions, each peptide precursor was fragmented again as for MS2 analysis followed by synchronous selection of the up to 8 most intense peptide fragments in the IT (18) and further fragmentation via HCD using a NCE of 55%. The MS3 scan was recorded in the OT at 50K resolution (scan range 100–1000 *m*/*z*, isolation window of 1.2 *m*/*z*, AGC of 1e5 charges, maxIT of 86 ms). The cycle time was 1.2 s and the dynamic exclusion lasted for 50 s.

##### SARS-CoV-2 Spectral Library Generation

High-quality spectral libraries for SARS-CoV-2 peptides were generated for both nano-flow and micro-flow systems using a synthetic SARS-CoV-2 peptide mix. Nano-flow DDA LC-ESI-MS/MS measurements were performed on a Dionex UltiMate 3000 RSLCnano System (Thermo Fisher Scientific). Synthetic peptides were dissolved in 0.1% FA in 2% ACN and PROCAL and iRT peptides were spiked for retention time calibration. Peptides were delivered to a trap column (75 μm × 2 cm, packed in-house with 5 μm C18 resin; Reprosil PUR AQ, Dr. Maisch) and washed using 0.1% FA at a flow rate of 5 μl/min for 10 min Subsequently, peptides were transferred to an analytical column (75 μm × 45 cm, packed in-house with 3 μm C18 resin; Reprosil Gold, Dr. Maisch) applying a flow rate of 300 nl/min and separated using a 50 min linear gradient from 4 to 32% nano-flow solvent B (0.1% FA and 5% DMSO in ACN) in A (0.1% FA in 5% DMSO). A NSI source was used. Full scan MS1 spectra were recorded in the OT from 360 to 1300 *m*/*z* at 60 k resolution using an AGC target value of 4e5 charges and a maxIT of 50 ms. The top 20 precursors were selected for fragmentation and MS2 spectra were acquired after HCD fragmentation with 30% NCE and using an isolation window of 1.3 m/z, AGC target value of 5e4, and maxIT of 22 ms. Dynamic exclusion was set to 20 s. For micro-flow measurements, no RT peptides were spiked. Measurements were performed using a 15 min gradient as specified above with following modifications: MS1 scan range was set from 360 to 1860 *m*/*z* and RF-lens level to 50%. MS2 spectra were recorded at 60 k resolution with a fixed first mass of 100 *m*/*z* and using a maxIT of 118 ms and an intensity threshold of 2e4 charges. Cycle time was set to 1.1 s and no dynamic exclusion was specified.

##### PRM LC–MS/MS Measurements

A more detailed description of targeted measurements including pre-runs for developing the final SARS-CoV-2 PRM assay can be found in the supplemental Methods. In brief, nano-flow PRM measurements were performed using a 50 min linear gradient as described above for the spectral library generation but operating the Fusion Lumos in PRM mode. Targeted MS2 spectra were acquired at 60 k resolution within 100–2000 *m*/*z*, after HCD with 30% NCE, and using an AGC target value of 4e5 charges, a maxIT of 118 ms and an isolation window of 1.3 *m*/*z*. The number of targeted precursors was adjusted to a cycle time of at maximum 2 s. For the PRM analysis of the dilution series samples and nasopharyngeal swab samples, 23 optimal SARS-CoV-2 peptide precursors plus 11 iRT peptide precursors were targeted within a single PRM measurement and with a 6 min scheduled retention time window. Micro-flow PRM measurements were performed using a 15 min linear gradient as described above. In contrast to targeted nano-flow measurements, targeted MS2 spectra were acquired after HCD with 32% NCE and using an AGC target value of 1e5 charges. The number of targeted precursors was adjusted to a cycle time of at maximum 0.9 s. In total, 21 SARS-CoV-2 peptides or 21/15 human/monkey ACE2 and TMPRSS2 peptides were targeted in 1 min wide transition windows except for peptides for which no experimental retention time was available (monkey TMPRSS2 peptides not mapping to the human sequence) in which case 2 min wide transition windows were employed. No peptides for retention time calibration were scheduled for fragmentation, but PROCAL peptides were spiked into samples to use MS1 chromatogram information.

##### DDA Database Searching

Peptide and protein identification and quantification for DDA type of experiments was performed using MaxQuant (v1.6.3.4) with its built-in search engine Andromeda (19). Tandem mass spectra derived from human cell lines were searched against the human reference proteome (UP000005640, 96,821 entries including isoforms, last modified on 15th of Jan 2020). For Vero E6 derived raw data, UniProtKB sequences for the taxonomy *Chlorocebus* (20,699 entries including isoforms, downloaded on May 12, 2020) and RefSeq sequences for the species *Chlorocebus sabaeus* (61,803 entries, downloaded on the May 4, 2020) were used. For the baseline proteomes of Vero E6 cells, *Chlorocebus* sequences were supplemented with the human reference proteome to enable proteomics-guided genome refinement. Spectra from SARS-CoV-2 containing samples were additionally searched against the UniProtKB SARS-CoV-2 proteome (UP000464024, 14 entries, last modified on March 22, 2020) supplemented with recently reported novel ORFs (20). Further, common contaminants and, where applicable, retention time peptides were added to all searches. For label-free samples, the experiment type was left in default settings, whereas 10plex TMT was specified as isobaric label within a reporter ion MS3 experiment type for TMT-labeled Vero E6 samples. Isotope impurities of the TMT lot (#TE268169) were specified to allow MaxQuant the automated correction of TMT intensities. The MaxQuant searches of the DDA data obtained from the isotopically labeled synthetic peptide mixes were performed by selecting Arg10 (C-terminal), Lys8 (C-terminal) and Lys7 (anywhere) as variable modifications. For all searches, carbamidomethylated cysteine was set as fixed modification and oxidation of methionine and N-terminal protein acetylation as variable modifications. Trypsin/P was specified as the proteolytic enzyme with up to two missed cleavage sites allowed and absolute quantification by iBAQ was enabled. Precursor tolerance was set to ±4.5 ppm and fragment ion tolerance to ±20 ppm and ±0.35 Da for OT and IT spectra, respectively. Matching was enabled only between fractions of the same proteome (5 min alignment window, 0.2 min matching window). Default score cutoffs were used requiring a minimal Andromeda score of 40 and a delta score of 6 for modified peptides. Results were adjusted to 1% peptide spectrum match and 1% protein false discovery rate (FDR) employing a target-decoy approach using reversed protein sequences. Protein quantification was obtained from the summed area under peptide elution profiles for label-free samples or from summed peptide reporter intensities for TMT-labeled samples.

##### Data Analysis

Details on the exemplary refinement of the gene annotation of the *Chlorocebus sabaeus* genome are specified in the supplemental Methods and the respective results sections.

##### Cell Line Full Proteomes

Absolute protein quantification was derived from iBAQ values (intensity-based absolute quantification of proteins, according to Schwanhausser *et al.* (21)) as provided by the MaxQuant software. Hits to the reverse and contaminant database were removed. To correct for different loading amounts of the four different cell lines and enable comparison of protein expression levels, iBAQ values were normalized by median centering. A list of 332 confident interactors published by Gordon *et al.* (2) was mapped to our cell line data based on gene names. To determine potential virus interactors that exhibited the most differential expression across the four cell lines, first the ratio of the iBAQ in one cell line to the median iBAQ across all four cell lines was calculated for each potential interactor. The top 5% of proteins showing the highest deviation from the median were categorized as differentially expressed. All analyses were performed using R (version: 3.6.0) and heat maps were plotted with the help of the R package pheatmap.

##### Virus-Dose Experiment

A more detailed description of the analysis of the SARS-CoV-2 infected Vero E6 cells can be found in the supplemental Methods. In brief, hits to the reverse and contaminant database were removed. Reporter ion intensities of multiplexed mock and SARS-CoV-2 treated Vero E6 samples were normalized for mixing errors based on the total sums of peptide intensities in each channel. The Perseus software suite (v.1.6.14.0) was used to perform correlation analysis, principal component analysis, two-sided Student's t-tests, clustering, Fisher's exact tests, and functional 2D enrichments (22). For comparison of our data with a published virus-host response study in Caco-2 (3), their provided supplemental data on expression changes 24 h post infection with SARS-CoV-2 at 1 MOI were re-analyzed for significantly changing proteins analogous to our data. All annotations were mapped to our data set based on gene names.

##### PRM Data Analysis

Details of the PRM data analysis are given in the supplemental Methods. In brief, nano-flow and micro-flow PRM data were analyzed using the Skyline-daily (64-bit) software (version 20.1.1.83) (14) and reviewing peak integration, transition interferences and integration boundaries manually. Five to seven transitions per peptide were considered. To discriminate between positive and negative ACE2 and TMPRSS2 peptide detection, filtering according to mass accuracy (max. ±4 ppm) and correlation of fragment ion intensities between the light (endogenous) SARS-CoV-2 peptide and the experimental library spectrum (“Library Dot Product” > 0.85) was applied. In the clinical cohorts, positive peptide detection additionally required a correlation of fragment ion intensities between the light and heavy (spike-in) peptide (“DotProductLightToHeavy”) of more than 0.9. Total protein or virus intensity per cell line or clinical sample was computed by summing up all light peptide intensities detected positive in each sample. Uniqueness of SARS-CoV-2 peptides was assessed against a nonredundant protein database including entries from GenPept, Swissprot, PIR, PDF, PDB, and RefSeq (downloaded on May 3, 2020 from https://ftp.ncbi.nlm.nih.gov/blast/db/FASTA/nr.gz).

## RESULTS

##### Proteomes of Cellular Model Systems for SARS-CoV-2 Research

**Fig. 1:**
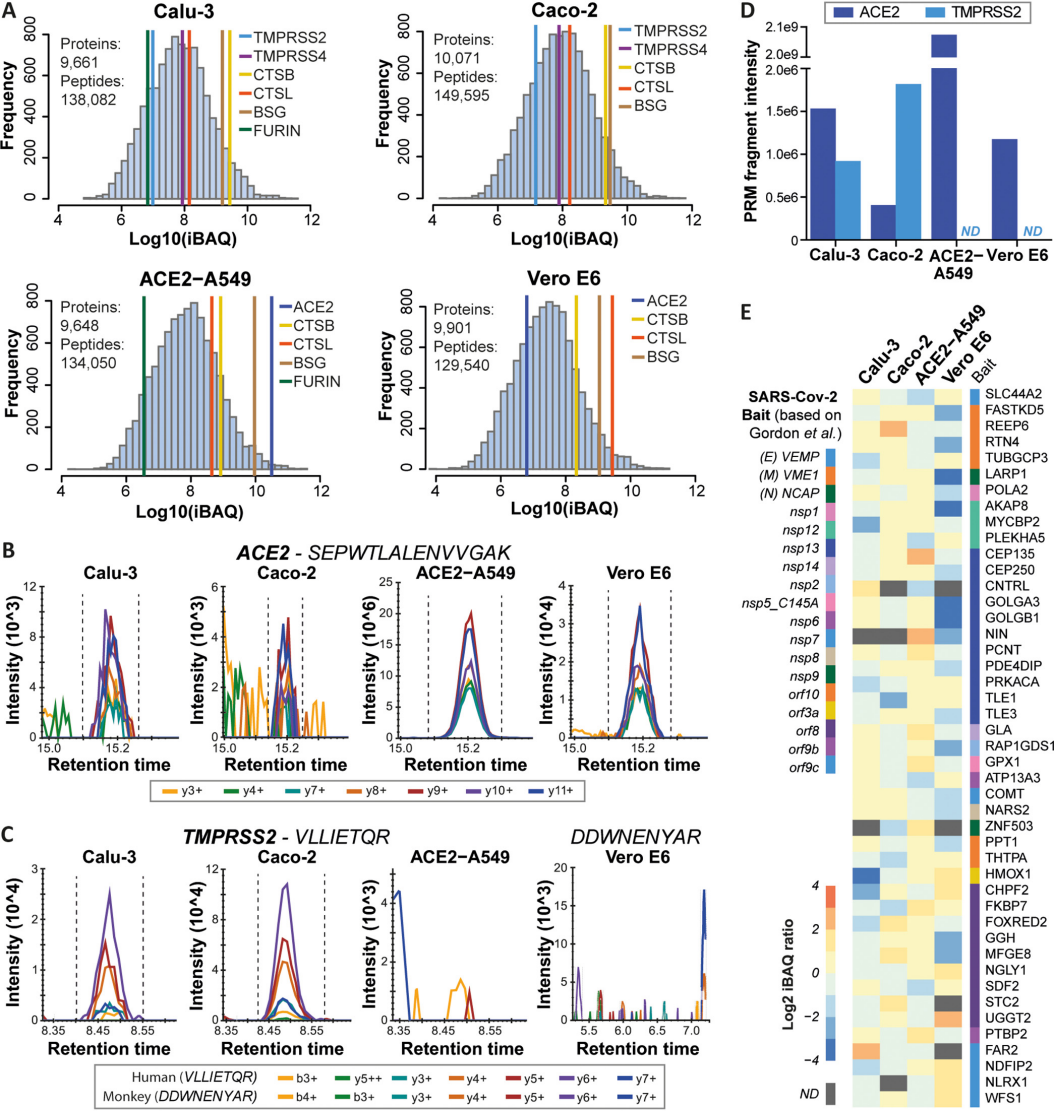
**Deep proteome profiling of four model cell lines used for SARS-CoV-2 research.***A*, Protein abundance distributions for human Calu-3, Caco-2, and ACE2-A549, and African Green Monkey Vero E6 cells. Abundances of proteins thought to be involved in viral entry into host cells are marked. *B*, PRM transitions for a peptide shared among human and monkey ACE2 in the four model cell lines. *C*, Same as (*B*) but for a human and a monkey TMPRSS2 peptide. *D*, Bar chart showing summed PRM-MS2 intensities of confidently detected peptides for ACE2 and TMPRSS2 in the four model cell lines. Please note that intensities for human and monkey ACE2 are not directly comparable because in part different peptides were targeted (ND: not detected). *E*, Heat map showing the abundance of 45 high-confidence interactors of SARS-CoV-2 proteins according to Gordon *et al.*, which are also differentially expressed across the four cell lines used in the current study (see supplemental Fig. S2B for details).

A number of cell lines are recurrently used for research on coronaviruses including the human epithelial lung cancer cell line Calu-3, the human epithelial colorectal adenocarcinoma cell line Caco-2, and the epithelial renal cell line Vero E6 established from the African green monkey (*Chlorocebus sabaeus*). We created a further cell line on the basis of the human alveolar basal epithelial adenocarcinoma cell line A549 that stably expresses HA-tagged human angiotensin-converting enzyme 2 (ACE2), a cell surface protein generally considered important for entry of coronaviruses into host cells (6). To characterize these model systems in terms of protein expression and to enable the construction of spectral libraries, we generated deep-scale proteomic profiles of these cell lines using a workflow consisting of SDS lysis and SP3 digestion on a robotic work station (10), off-line peptide separation into 48 fractions using high pH reversed phase chromatography, online analysis of each fraction using 15 min gradients on a micro-flow LC (17) coupled to an Orbitrap Lumos instrument with high-resolution precursor and fragment ion detection and protein quantification by the iBAQ approach (21). After filtering the data for 1% peptide and protein false discovery rate (FDR), a total of 9,661, 10,071, 9,648 and 9,901 proteins (136,082, 149,595, 134,050, 129,540 peptides) were identified for Calu-3, Caco-2, ACE2-A549 and Vero E6 cells respectively (Fig. 1*A*; supplemental Table S1). Workflow replicates analyzed by ion trap MS2 showed good correlation of MS1 intensities (*r* = 0.88 for ACE2-A549; *r* = 0.72 for Vero E6) and comparable figures for protein and peptide identifications (10,289/165,745 for ACE2-A549 and 9905/128,980 for Vero E6; supplemental Fig. S1A, S1B).

Even though the protein expression data measured by data dependent acquisition (DDA) covers six orders of magnitude of dynamic range, ACE2 could be identified only in ACE2-A549 and Vero E6 cells but not in Calu-3 and Caco-2 cells. The protein could be detected in the latter two cell lines by parallel reaction monitoring (PRM; not by western blotting) but their levels were >1000 lower than in ACE2-A549 cells (Fig. 1*B*–1*D*; supplemental Fig. S1C; supplemental Table S1). A similar observation was made for TMPRSS2, which was not detected in ACE2-A549 and Vero E6 cells by DDA or PRM. Other proteins discussed as viral entry factors such as TMPRSS4, CTSB, CTSL, BSG and FURIN (23, 24, 25, 26) also showed substantial differences in expression levels between cell lines. The apparent lack of co-expression of ACE2 and TMPRSS2 in ACE2-A549 and Vero E6 was unexpected as both proteins have been reported to act in concert to facilitate viral entry.

The cell line proteomes also cover 311 of the 332 high-confidence interactors of a recently published SARS-CoV-2 protein interactome performed in HEK293 cells (2). Most of these proteins showed rather uniform expression patterns between cell lines, but some differed more than 10-fold in abundance (*e.g.* HMOX1, GOLGA3; Fig. 1*E*; supplemental Fig. S2A, S2B; supplemental Table S1). Therefore, one might expect that viral interactomes may also differ somewhat between cell types. In our laboratory, Vero E6 and ACE2-A549 cells were readily infectable by a SARS-CoV-2 GFP-reporter virus (27). Caco-2 cells showed much less viral susceptibility and no productive infection could be obtained for Calu-3 cells (supplemental Fig. S2C). The ability to infect different host cells depends on many factors and our baseline proteomes may become useful resources for addressing this question by more specialized experiments.

##### Proteomic Annotation of the Chlorocebus Genome in Vero E6 Cells

Most proteomic researchers rely on Uniprot and, to a lesser extent, on RefSeq for the provision of high-quality, annotated protein sequences. Both resources currently contain ∼20,000 entries for *Chlorocebus sabaeus,* only six of which have been reviewed by Uniprot. Instead, almost all annotated sequences are predictions based on the published *Chlorocebus sabaeus* genome (28). The Vero E6 genome has not been sequenced yet. Published genome information is available for a related cell line (Vero JCRB0111) (29), but no annotated list of protein-coding regions is included. To investigate which *Chlorocebus* genes exist as proteins and to examine if orthologs of human genes may have been missed or only partially found, we searched the >600,000 high-resolution tandem mass spectra collected from Vero E6 protein digests against protein sequences from Uniprot_Chlorocebus, RefSeq_Sabeus and Uniprot_Human. This led to the identification of 9840 proteins represented by 127,669 peptides that mapped to *Chlorocebus* sequences providing clear protein level evidence for the transcription and translation of the underlying genes (supplemental Fig. S3A). This proteomic coverage is like the ones obtained for the human cell lines indicating that the overall annotation of the *Chlorocebus* genome for protein-coding regions is already quite good. However, the search also identified 61 proteins and 1871 peptides that exclusively mapped to human sequences (supplemental Fig. S3A). Some of these may be trivially explained by false matches to the human database given the 1% FDR filters applied, notably proteins supported by one or two peptides only. However, several human-only proteins were identified with many peptides (in one case 29 peptides; supplemental Fig. S3B). Also, the list contained many human-only peptides with very high Andromeda scores and strong MS1 intensities, and their distributions were identical to the shared peptides (supplemental Fig. S3C, S3D) indicating that these matches should be trustworthy and indeed represent *Chlorocebus* encoded proteins. Such peptides may, therefore, cover genes or exons that were overlooked during *Chlorocebus* genome annotation, result from mistakes in splicing of the predicted monkey exons *in silico*, represent single nucleotide variants (SNVs) in the genes of the Vero E6 cell culture used in this study, or reflect errors in monkey genome sequencing or assembly.

**Fig. 2:**
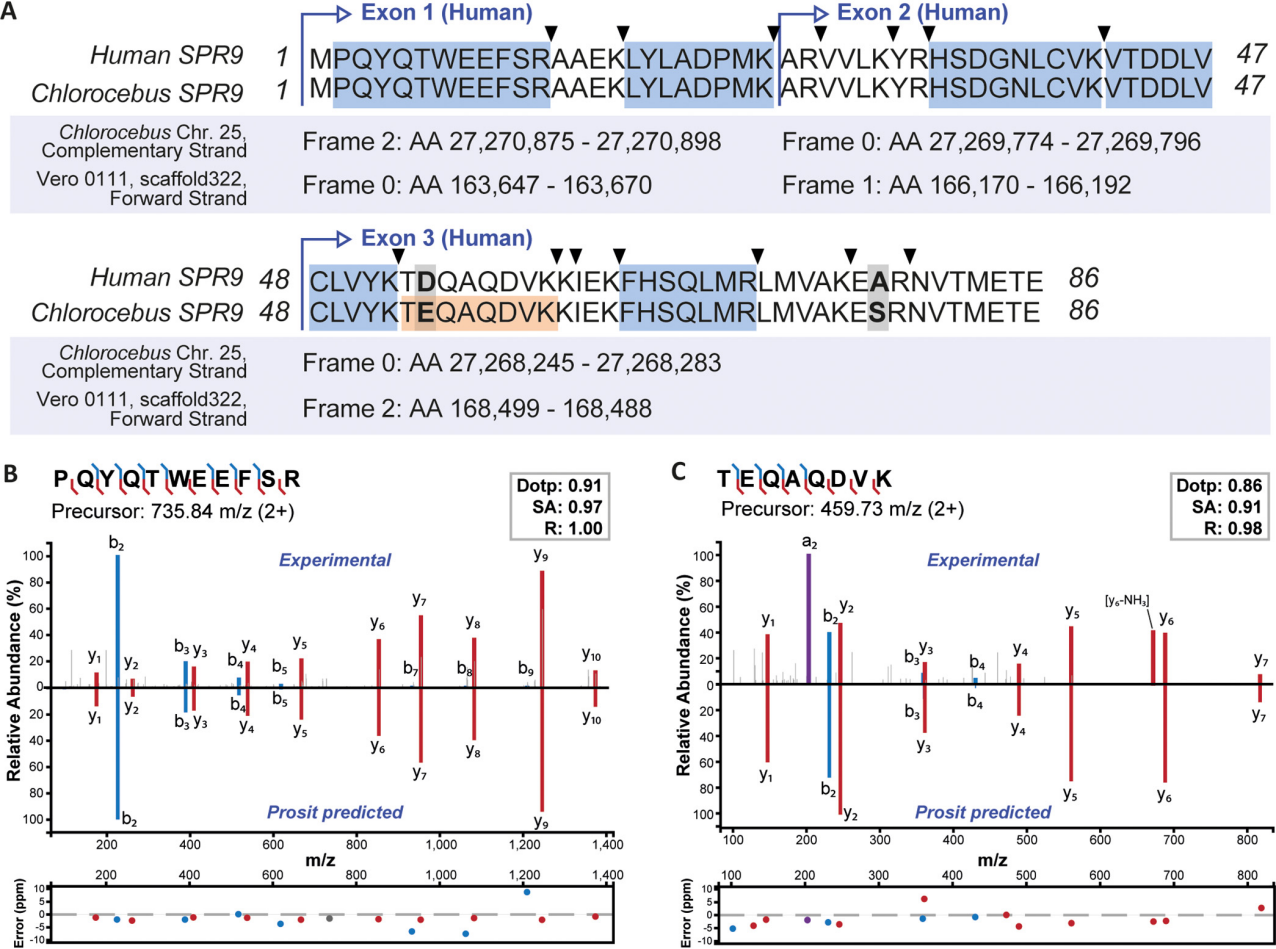
**Proteomics-guided annotation of a missed gene in the *Chlorocebus* genome.***A*, Alignment of the human SRP9 protein sequence with its *Chlorocebus* ortholog constructed from a six-frame translation of the genomic *Chlorocebus* and Vero JCRB0111 (Vero 0111) sequences. Peptides in blue map to all, the human, the *Chlorocebus,* and the Vero JCRB0111 sequences. The peptide coloured in orange is unique for *Chlorocebus* and Vero JCRB0111 and was only identified in a refined search including the newly annotated monkey SRP9 sequence. Triangles indicate trypsin cleavage sites and bold-face letters mark amino acid differences between the sequences. *B*, Mirror and m/z deviation plots of the experimental spectrum and the Prosit predicted spectrum for the N-terminal peptide of the human and *Chlorocebus* protein sequence. The similarity of the two spectra is measured by the dot product (dotp), the spectral contrast angle (SA) and the Pearson correlation coefficient (R) of the two spectra. *C*, Same as in (*B*) but for the peptide that is unique for the *Chlorocebus* and Vero JCRB0111 sequences of SRP9.

An example for a missed gene is SRP9 (signal recognition particle 9 kDa protein), a small protein involved in targeting secretory proteins to the rough endoplasmic reticulum membrane. The database search identified five peptides that matched the human sequence but none identified a *Chlorocebus* sequence in Uniprot, RefSeq or a list of predicted proteins for the Vero JCRB0111 cell line (kindly provided by Naoki Osada and Kentaro Hanada). A search of these peptides against six-frame translations of the *Chlorocebus* or Vero JCRB0111 genomes identified all five peptides. We then aligned the human protein sequence with the 6-frame translations to generate a new gene model for this protein (Fig. 2*A*), which is 98% identical to the human sequence (100% identity between *Chlorocebus* and Vero JCRB0111). Adding this newly predicted protein to the *Chlorocebus* fasta file and searching the LC–MS/MS data again led to the identification of one additional peptide that contains a Glu at position 54 in *Chlorocebus* instead of an Asp in human. The validity of peptide assignments was confirmed by mirror mass spectra comparing the experimentally obtained tandem MS spectrum to that predicted by the artificial intelligence Prosit (15) (Fig. 2*B*, 2*C*).

We also identified an example for overlooked exons represented by the breast cancer type 1 susceptibility protein (BRCA1, supplemental Fig. S4). Peptide hits to the human sequence indicated that the large exon 9 was missing in the deposited *Chlorocebus* protein sequence, whereas the small exons 2, 4, 5, 6, and 7 were absent in the Vero JCRB0111 protein sequence. Refining the gene models as described above resulted in 41 peptides that mapped along the entire *Chlorocebus*/Vero BRCA1 sequence and identified one peptide that was specific for Vero JCRB0111 and one for *Chlorocebus*. Together, the above analysis clearly shows that the human and Green monkey BRCA1 proteins are of similar size and highly related to each other. Although not systematically investigated here, there are likely further examples of single amino acid variants (SAAVs), missing exons or missing genes in the data set. Hence, a future extension of this work could be the searching of the provided high-quality LC–MS/MS data collected for the Vero E6 cell line against six-frame translations of the entire *Chlorocebus* or Vero cell line genomes. This would enable a more systematic proteogenomic annotation of the African green monkey genome.

##### Proteomic Response of Vero E6 Cells on Infection with SARS-CoV-2

Viruses turn host cells into virus producing factories. Although the Vero E6 cell line is not a physiological model, it is used frequently in virus research. Their impairment in proper antiviral immunity because of *e.g.* their intrinsic deficiency in mounting an appropriate interferon response (owing to a 9-Mb deletion on chromosome 12 that contains the type I interferon gene cluster (29)) makes Vero E6 cells applicable to studying many different viruses. To generate proteome-wide information on a cell line that contains the cellular machinery required for highly efficient virus growth, we infected Vero E6 cells with SARS-CoV-2 using two different doses (mock, MOI of 0.1 and 3) and collected the proteomes 24 h post infection (each in triplicate). Digests of all samples were labeled by tandem mass tags (TMT), combined into a TMT-9-plex peptide pool, and processed as described above. Relative peptide quantification was performed using the synchronous precursor selection (SPS)-based MS3 approach (18). This led to the highly reproducible (Pearson R > 0.98 for all TMT channels) identification and quantification of 7,287 proteins in at least two replicates of each condition supported by 68,778 peptides (supplemental Fig. S5A, S5B, supplemental Table S2).

**Fig. 3:**
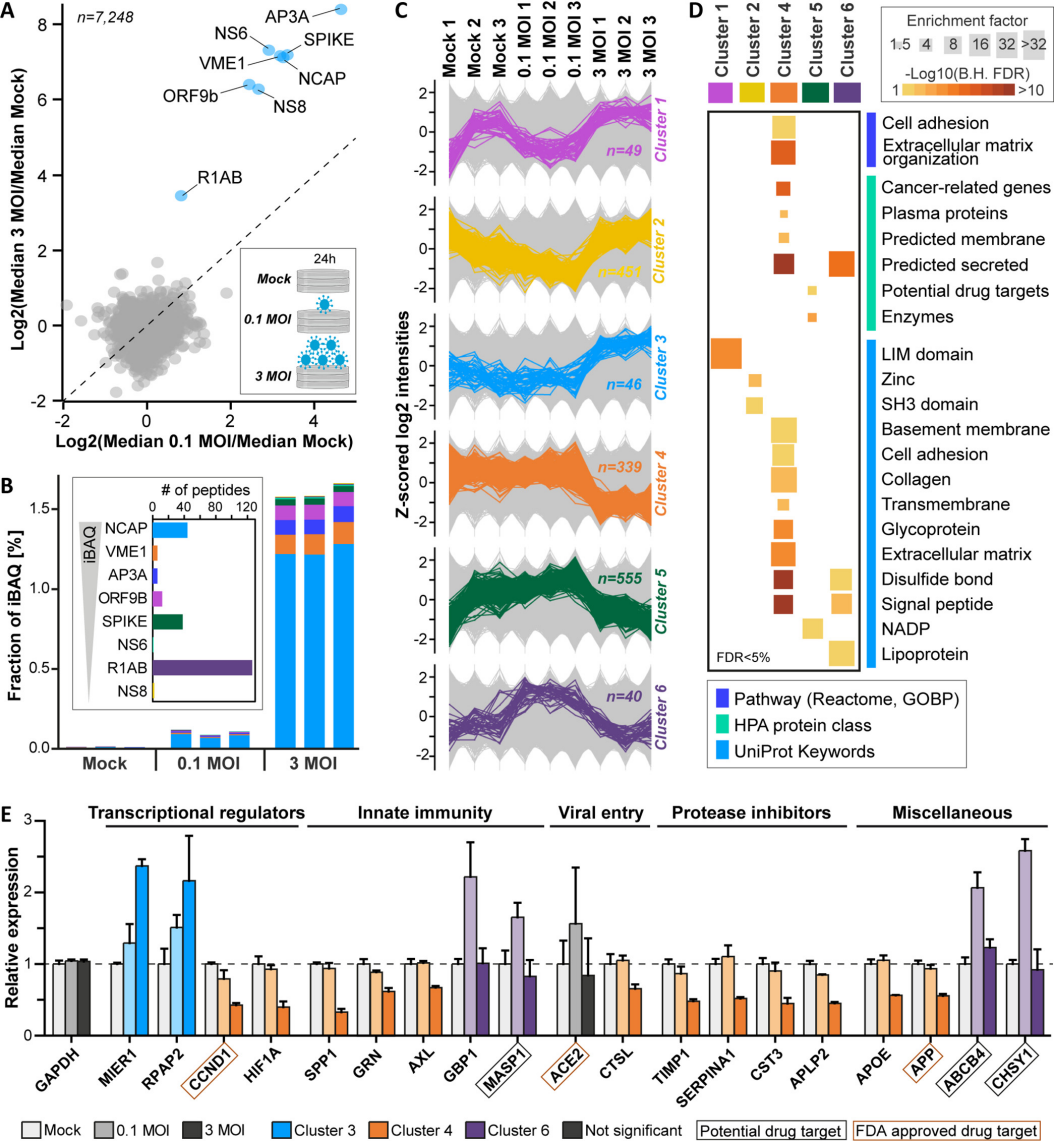
**Vero E6 proteome response after infection with SARS-CoV-2. Vero E6 cells were infected with SARS-CoV-2 at 0.1 MOI, 3 MOI and mock in triplicate and proteomes were profiled 24h post infection.***A*, Protein expression changes for 3 MOI *versus* 0.1 MOI. Annotated viral proteins are marked in blue. *B*, Bar chart showing the fractional abundance of viral proteins in the host cell proteome. The inset displays the number of identified peptides per virus protein ordered by decreasing cellular abundance. *C*, Line charts illustrating the expression patterns of proteins in the six main clusters extracted from significantly regulated proteins. Background proteins are displayed in grey. *D*, Functional categories enriched in clusters determined by Fisher's exact tests (B.H. FDR: Benjamini-Hochberg false discovery rate). *E*, Examples for regulated proteins from different clusters. The dotted line indicates no change (exemplified by GAPDH).

As one would expect, viral proteins were strongly expressed 24 h post infection (Fig. 3*A*). We detected 8 of the canonical viral proteins but found no evidence for expression of alternative proteins predicted by a recent study (20). The detected viral proteins made up more than 1.5% of the total Vero E6 proteome with the nucleoprotein (NCAP) being by far the most abundant protein followed by the membrane protein (VME1), the ion channel viroporin (accessory protein 3a, AP3A), the spike glycoprotein (SPIKE) and the protein 9b (ORF9B; Fig. 3*B*). Despite the detection of a total of 126 distinct peptides, the replicase polyprotein 1ab (R1AB) was of lower abundance than the other viral proteins but, with a median log10 iBAQ value of 7.0, it was still much higher expressed than the median of all Vero E6 proteins (iBAQ of 6.2). The data also clearly shows that a higher inoculation dose led to a much stronger expression of all viral proteins but with similar relative levels between conditions (Fig. 3*B*; supplemental Fig. S5C). We observed statistically significant expression changes of ∼1500 host cell proteins and grouped them into 6 clusters according to a consistent expression behavior between the three experimental conditions (Fig. 3*C*; supplemental Fig. S5D–S5G; supplemental Table S2). In all but cluster 3, we found a statistically significant enrichment of ontologies (Fig. 3*D*; supplemental Fig. S6A) and further proteins with noteworthy functional annotations (Fig. 3*E*; supplemental Fig. S6B).

Among significantly changing proteins, several transcriptional regulators showed up- or down-regulation on SARS-CoV-2 infection. For instance, the transcriptional repressor MIER1 and the phosphatase RPAP2, which promotes transcription of snRNA genes by regulating the activity of the RNA polymerase II subunit POLR2A, were among the proteins exhibiting the strongest virus dose-dependent up-regulation. In contrast, the G1/S specific cyclin CCND1 and the transcription factor HIF1A – together with several other cell cycle regulators and mediators of oxidative stress – were strongly down-regulated at high viral loads (supplemental Fig. S6B). The expected negative impact of viral infection on Vero E6 cell proliferation was apparent by the fact that cell confluence was decreased in a virus dose- and infection time-dependent fashion (supplemental Fig. S7). Further, we observed a decrease in expression of several proteins involved in innate immunity such as the cytokine SPP1, the growth factor GRN, and the receptor tyrosine kinase AXL (in total 22 down-regulated, innate immunity-associated proteins according to Reactome annotations; supplemental Table S2). In contrast, the GTPase GBP1, which has been described to exhibit antiviral activity, and MASP1, a serine endopeptidase and critical component of the complement system, were up-regulated only at low viral inoculation (Fig. 3*E*). Moreover, expression changes of additional proteases as well as protease inhibitors, which often also exhibit immune-modulatory functions and are, in part, involved in blood coagulation, were frequently observed. As an example, although ACE2 levels did not change significantly, the expression of CTSL, another protein potentially involved in SARS-CoV-2 entry, was down-regulated. The same applied to many protease inhibitors such as the metalloproteinase inhibitors TIMP1/2, the serine protease inhibitors alpha-1-antitrypsin (SERPINA1) and APLP2, and the cysteine protease inhibitor CST3. Other groups of regulated proteins were implicated in lipid homeostasis, vesicle trafficking, glycosylation, and cell adhesion and contained, for instance, the apolipoprotein APOE, the phosphatidylcholine translocator ABCB4, the cholesterol transporter GRAMD1A, the GTPase-activating protein RALGAPA1, the H+/Cl- exchange transporter CLCN3, the chondroitin sulfate synthase CHSY1, and the collagens COL6A2, 10A1, and 18A (supplemental Fig. S6B and supplemental Table S2).

**Fig. 4:**
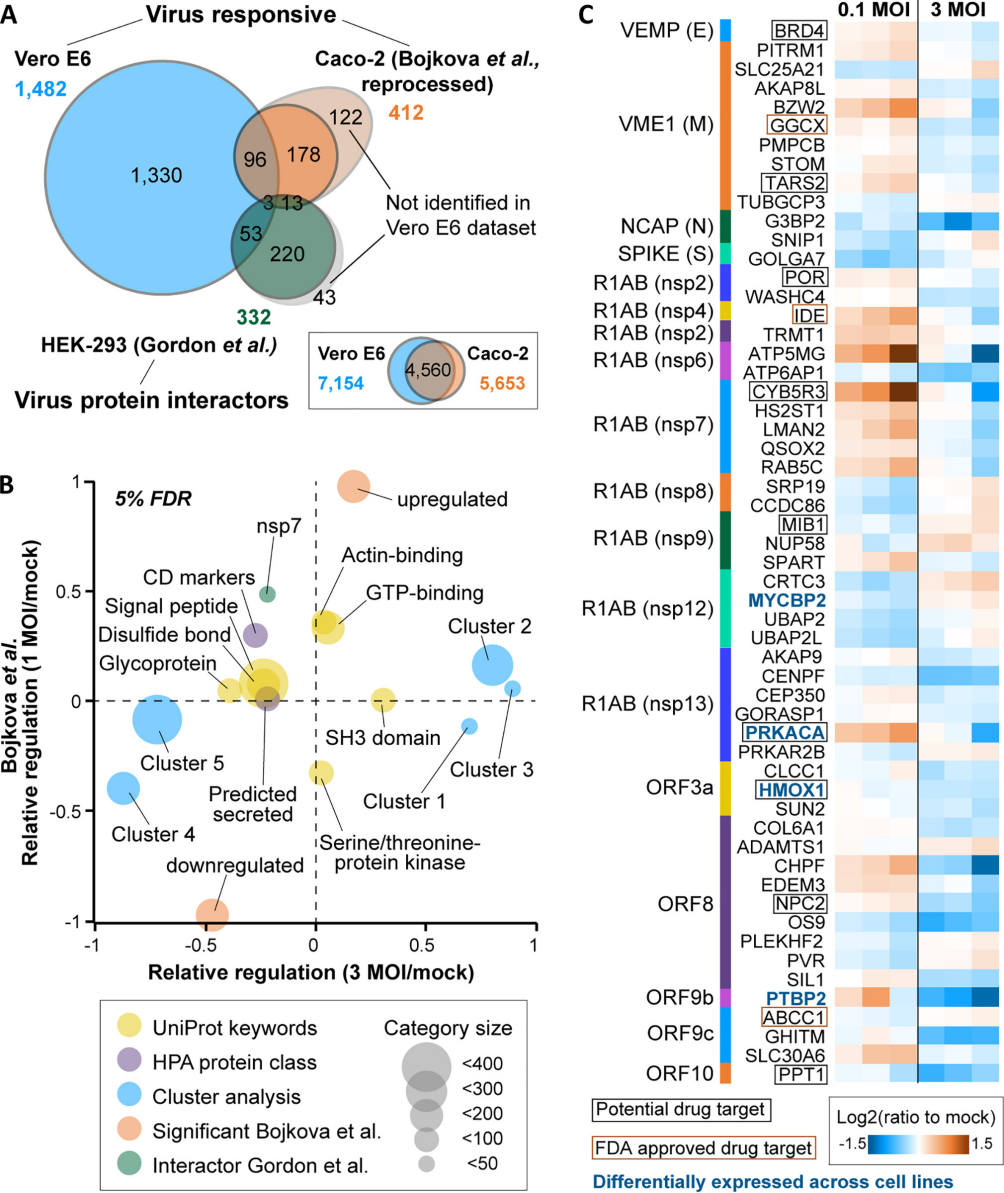
**Comparison of the Vero E6 response to SARS-CoV-2 to a recently published Caco-2 infectome and a SARS-CoV-2 interactome.***A*, Venn diagram illustrating the overlap of virusregulated proteins (Vero E6, Caco-2) and high-confidence SARS-CoV-2 interactors (HEK-293). The inset shows the overlap of all quantified gene products in the two infectome studies. *B*, 2D enrichment analysis displaying annotation terms whose members show consistent or divergent behaviour in the two infectomes. Only categories with a |relative regulation| >0.2 in at least one of the two datasets are displayed. *C*, Heatmap showing the 56 proteins that were significantly regulated in the Vero E6 infectome and reported as high-confidence interactors of SARS-CoV-2 proteins.

Next, we aligned the Vero E6 infectome generated in this study with a conceptually similar recent experiment performed by Bojkova *et al.* in Caco-2 cells, in which some 1,500 regulated proteins 24 h post SARS-CoV-2 infection were reported (3). To be able to compare significantly regulated proteins in the two infectomes, we re-processed the Bojkova *et al.* data using the same statistical criteria applied to ours (Fig. 4*A*). Given the substantial experimental differences between the two studies (*e.g.* cell line, virus load, pulsed SILAC *versus* TMT quantification), a 2-dimensional enrichment analysis showed only partially consistent results in that proteins that were up- or down-regulated in one study were generally also up- or down-regulated in the other (Fig. 4*B*; supplemental Fig. S8A–S8C). As mentioned above, the Vero E6 cell line is not a physiological model, so the substantial differences in results suggest cell line-characteristic responses. As a result, more general conclusions may be drawn only by performing further such experiments in the future and using more physiologically relevant model systems.

A comparison of our infectome with the aforementioned SARS-CoV-2 interactome performed by Gordon *et al.* in HEK293 cells (2) revealed that 56 interacting host proteins showed quantitative changes in the Vero E6 infectome (Fig. 4*C*; supplemental Fig. S8D). These proteins represent a multitude of different functions, and most showed small, albeit significant regulations. However, several FDA approved drug targets or proteins with active drug discovery programs exhibited larger regulations (*e.g.* CYB5R3). Some of these were also differentially expressed across our four model cell lines (*e.g.* PRKACA), supporting the notion of, and providing some initial hypothesis for cell line-specific effects during SARS-CoV-2 infection.

##### Detecting SARS-CoV-2 Proteins by Mass Spectrometry

As shown above, 8 of the 14 predicted proteins in the SARS-CoV-2 reference proteome could be detected in Vero E6 cell digests, notably the virus polyprotein (R1AB), the viroporin ion channel (AP3A), the spike glycoprotein (SPIKE), the viral membrane protein VME1, the nonstructural proteins NS6 and NS8, the nucleoprotein NCAP, and the alternative ORF9b (Fig. 3*A*). We next analyzed the supernatant of virus producing Vero E6 cells to investigate which viral proteins may be identified from an assembled (perhaps not always fully or functional) virus. Because cell supernatants contain substantial quantities of proteins, metabolites and other components from the cell culture medium, we first compared three sample preparation techniques, notably the aforementioned SP3 approach, an in-solution protocol using urea as a chaotrope and the classical in-gel digestion protocol. These data are summarized in supplemental Fig. S9 and show that all gave qualitatively similar results, but with differences in detail. In total, five viral proteins were detected in cell culture supernatants (SPIKE, VME1, NS8, NCAP and ORF9b). As expected, the viral replicase and the viroporin ion channel detected in cells could not be found in the supernatant as these are not part of the assembled virus. All three protocols robustly detected NCAP. The protocols involving SDS denaturation (in-gel and SP3) better extracted the membrane proteins SPIKE and VME1 and gave fewer missed-cleaved peptides than the urea protocol. The in-gel and SP3 protocols were not as efficient for ORF9b, and the nonstructural protein NS8 was generally not well detected. Differences in the sample preparation protocols were more obvious at the peptide level, but replicate analysis showed that each protocol yielded consistent results in terms of MS signal intensity of the identified peptides.

##### Parallel Reaction Monitoring (PRM) Assays for SARS-CoV-2 Proteins

**Fig. 5:**
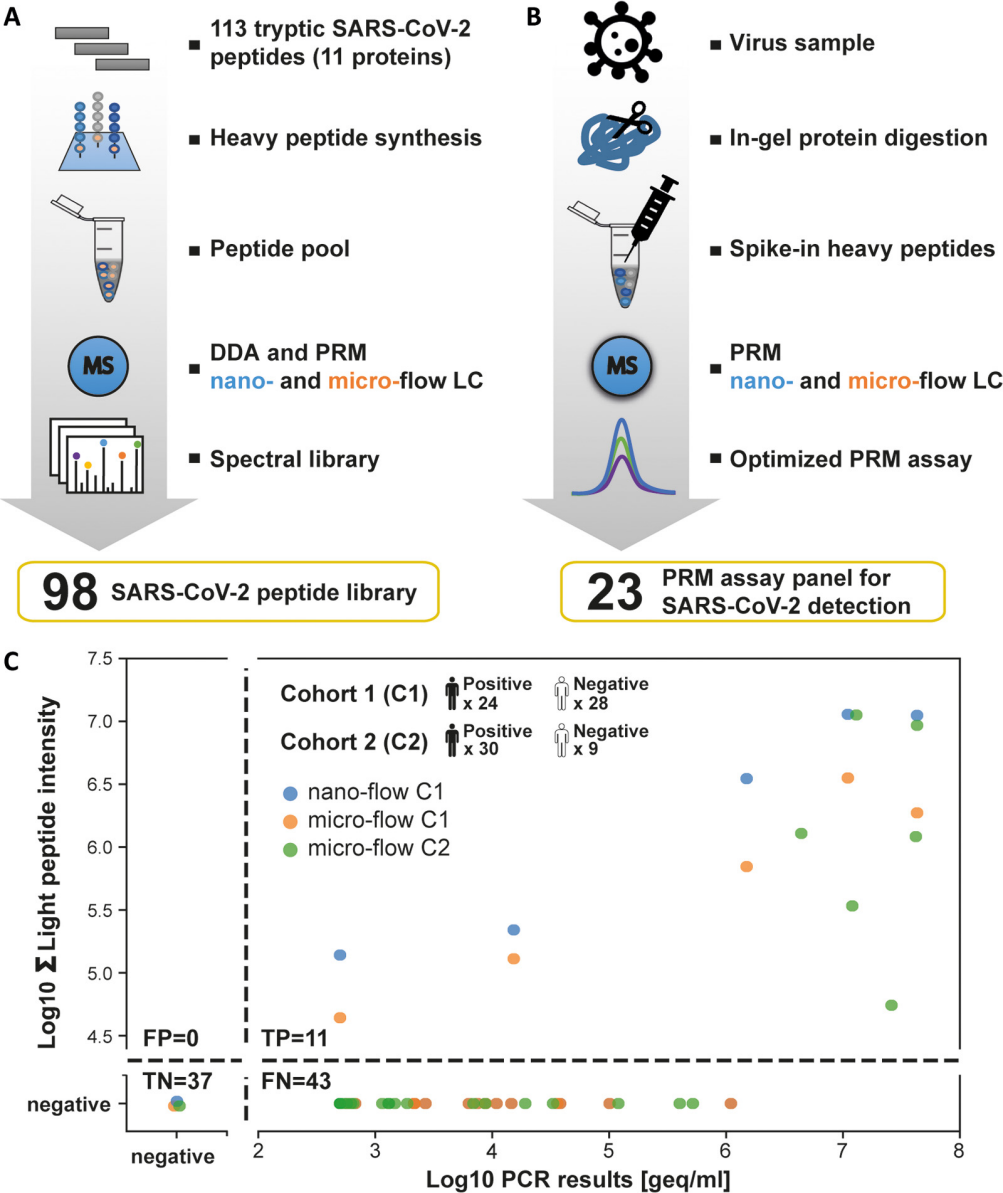
**PRM assays for SARS-CoV-2 detection in clinical samples.***A*, Flow chart illustrating the generation and characterization of a synthetic SARS-CoV-2 peptide and spectral library. *B*, Flow chart for the optimization of a PRM assay panel for SARS-CoV-2 detection. *C*, Comparison of SARS-CoV-2 detection in clinical samples by PCR and PRM. Two cohorts (C1 and C2) of in total 54 PCR-positive and 37 PCR-negative diagnostic samples were analyzed. The semi-quantitative data of the PRM (summed endogenous (light) intensity) and PCR (genome equivalents, geq) assays are plotted against each other. The number of false positive (FP), true positive (TP), true negative (TN), and false negative (FN) cases were computed based on the PCR data as ground truth. Cohort 1 was measured by nano- and micro-flow PRM. Cohort 2 was only analyzed by micro-flow PRM.

Given the many biological sources from which samples of COVID-19 suspected individuals are taken (nasopharyngeal swab, bronchoalveolar lavage, blood, stool etc.) and that these sources themselves represent complex proteomes, we reasoned that PRM assays including stable isotope-labeled spike-in peptides would be required for the unambiguous identification of viral proteins in clinical samples. Figs. 5*A* and 5*B* illustrate the two-step process for the development and optimization of these assays. In the first step, we synthesized 113 stable isotope-labeled (heavy) peptides that represent all theoretical tryptic (and C-terminal) SARS-CoV-2 peptides with a length constraint between 7 to 24 amino acids covering 11 SARS-CoV-2 proteins (supplemental Table S3). DDA and PRM MS confidently detected 98 of these peptides (supplemental Fig. S10A; supplemental Table S3). Skyline (14) was used to build experimental spectral libraries from generated DDA as well as PRM data processed with MaxQuant (19). In addition, we also predicted a spectral library using the artificial intelligence Prosit (15).

In the second step of the PRM assay development, we used supernatants of infected Vero E6 cells to generate a panel of optimized PRM assays for endogenous SARS-CoV-2 protein detection (Fig. 5*B*). For the reasons of proteome complexity mentioned above, we opted for the in-gel digestion procedure. Although this, to some extent, sacrifices sample throughput, it is more robust against unknown factors, which turned out to be important for the analysis of COVID-19 suspected individuals (see further below). The in-gel digested supernatant sample was spiked with the heavy reference peptide pool and analyzed by PRM on the nano-flow LC–MS/MS system. Of the 98 targeted peptides, 57 endogenous virus peptides representing 5 proteins (SPIKE, NCAP, VME1, ORF9B, NS8) could be detected (supplemental Table S3).

To be able to perform PRM measurements on clinical material at a reasonable throughput, we further prioritized the list of targeted peptides for best MS2 signal and uniqueness to SARS-CoV-2. The latter is important in order not to confuse SARS-CoV-2 with other coronaviruses such as the four human endemic coronaviruses (229E, HKU1, NL63, and OC43) that are often the cause of the common cold. Perhaps surprisingly, only 60 of the 551 tryptic peptides of the 14 SARS-CoV-2 proteins were unique when searched against a tryptic *in silico* digest of several sequence collections (all species; supplemental Table S3). In addition, many of these were not well detectable by PRM. This led to the selection of 23 peptides for the final PRM assay (6 unique to SARS-CoV-2, supplemental Fig. S10B, supplemental Table S3; see supplement for further information). To characterize the PRM assays further, we in-gel digested dilutions of Vero E6 supernatant, spiked reference peptides at a constant concentration, and analyzed the samples by PRM (for an example see supplemental Fig. S11). The summed PRM signal and number of peptides supporting the detection of a viral protein rapidly declined for total protein starting amounts of <300 ng (micro-flow) and <50 ng (nano-flow), respectively. In both cases, the peptide VAGDSGFAAYSR (VME1) showed the best overall performance and marked the detection limit of the PRM assay (supplemental Table S3). IBAQ analysis of DDA data for the undiluted supernatant digest estimated that 75% of the protein in the sample is BSA, 17% correspond to Vero E6 proteins, 8% are spiked standards and only ∼0.4% are viral proteins. The latter underscores the very high sensitivity of the PRM assays.

##### Testing Clinical Samples for SARS-CoV-2 Infection Using PRM Assays

With the PRM assay in hand, we tested its merits on two diagnostic cohorts of 91 COVID-19 suspects (24 + 30 positive and 28 + 9 negative by PCR; supplemental Table S4). The viral loads determined by PCR spanned six orders of magnitude ranging from ∼30 to 43 million geq/ml (median 2400 geq). As one might expect from the way samples are collected, Coomassie blue staining of short SDS-PAGE gels showed large differences in the total amount of protein contained in each sample (supplemental Fig. S12).

The first cohort was measured by nano-flow and micro-flow PRM. For the latter, we used the same amount of starting material (by volume) as was used for the PCR test. For the former, only 10% of this quantity could be analyzed to make sure the trap column of the nano-LC was not overloaded. Of the 24 PCR-positive cases in the first cohort, five were also positive by PRM on either LC–MS/MS system (Fig. 5*C*). All PCR-negative cases were also negative by PRM. As the nano-flow system did not identify more positive cases, the second cohort was only analyzed by micro-flow PRM that is robust against differences in sample loading and only requires 20 min total analysis time compared with 70 min on our nano-flow system. Of the 30 PCR-positive cases, six were also positive by PRM and all PCR-negative cases were also negative by PRM (Fig. 5*C*). Loading even more material onto the micro-flow system did not improve the results further (not shown). The PRM-positive cases contained all 9 samples with >1 million geq/ml by PCR, one case with 15,000 geq/ml and one with <500 geq/ml (supplemental Fig. S12; supplemental Table S4). Most of the PRM-positive cases were detected by peptides representing the NCAP protein, but peptides from VME1, SPIKE and NS8 were also detected. When taking both cohorts together, only 11/54 (20%) of all PCR-positive cases could be detected by PRM demonstrating that the successful detection of SARS-CoV-2 in humans was generally confined to individuals with very high viral loads. In addition, when restricting a positive diagnosis to samples in which a unique SARS-CoV-2 peptide was detected, the success of the PRM went down to 7 cases (13%). The two PRM-positive cases with lower PCR values are puzzling because they appear out of range compared with the other PRM-positive cases. The detected peptide in these two samples is the one that showed the best PRM limit of detection in cell supernatant dilutions but is not unique to SARS-CoV-2. As the PRM data itself is of good quality, this potentially indicates a cross-reactivity of the PCR test with other coronaviruses.

## DISCUSSION

This work could not possibly address the merits of all conceivable applications of proteomics for SARS-CoV-2 research and testing, but a number of noteworthy observations from the current data, past experience and recent reports can be made, and these may project to other applications not covered here. For the general lack of peer-reviewed literature on SARS-CoV-2, frequent reference is made to manuscripts on preprint servers, which should be treated with the appropriate level of caution.

First, proteomic technology has progressed to a stage of technical development at which the proteomic characterization of biological model systems is a straightforward process. Here, we report on the proteomic landscape of four cellular model systems for SARS-CoV-2 research. Such baseline profiles are generally useful resources as they provide protein-level information of gene expression that defines the cell type and its phenotype on a molecular level and against which all observations made by specific experimentation can be gauged. The underlying mass spectrometric data can be used for constructing cell type-specific spectral libraries for *e.g.* DIA applications or targeted assays such as PRM/MRM. Exemplified by proteins discussed as viral entry factors, it is interesting to note that their expression varies greatly between cell types. More specifically, ACE2 and TMPRSS2 are generally regarded to be part of an important cell surface receptor system that mediates SARS-CoV-2 entry into host cells (6). Two recent reports showed ACE2 and TMPRSS2 co-expression in cells of the olfactory epithelium, the place where often very high virus loads can be detected (23, 30), and there are increasing reports on other viral entry ports such as the gut, where ACE2 is indeed expressed (31). However, our cell line proteome profiling data did not provide strong evidence for strict co-expression of the two proteins. By analyzing protein expression data available in ProteomicsDB (32, 33) (https://www.proteomicsdb.org), it becomes apparent that, although BSG and proteases like cathepsin L and B are expressed in many tissues and cell lines, FURIN, TMPRSS2/4, and ACE2 are not. ACE2 and TMPRSS2 can be found in several tissues but – interestingly – apparently not in the lung, the organ in which COVID-19 most often manifests as a severe disease. It is possible but not very likely that this is merely owing to insufficient sensitivity of MS, because the very extensive collection of antibody stains of human tissues in the Human Protein Atlas also shows no expression of ACE2 in the lung (34). This is mirrored by extensive gene expression data collected for airway epithelial cells and lung tissue (35). Together, this may suggest that the undoubtedly important ACE2-TMPRSS2 axis may not be the only receptor system by which SARS-CoV-2 can enter human cells.

Second, the fact that the MS data collected for Vero E6 cells identified nearly 10,000 proteins when searching against *Chlorocebus* sequences deposited in Uniprot suggested that the African green monkey genome appears to be overall well annotated for protein-coding regions. Hence, we were surprised to find that a described small human protein (SRP9) was entirely missed. Moreover, in this case, the order of the exons was reversed, which is hard to rationalize and suggests a mistake in genome assembly. Although small open reading frames can be difficult to find by automated gene finding algorithms, even more surprising was the finding that a large exon representing half the size of the *Chlorocebus* ortholog of human BCRA1 was not annotated in the Uniprot *Chlorocebus* proteome. We did not attempt to identify entirely new genes by searching 6-frame translations of the *Chlorocebus* genome using all of the LC–MS/MS data, but the high-quality data provided in this study could be used by interested parties to do just that. We note that great care must be taken in such proteogenomic investigations because searching large sequence spaces are plagued with false discoveries particularly for SAAVs. Still, our findings may motivate colleagues at Uniprot or RefSeq to refine the *Chlorocebus sabaeus* genome/proteome further and perhaps use the opportunity provided by our LC–MS/MS data to generate protein-level evidence for genes that only exist in *Chlorocebus*.

Third, a rather obvious proteomic experiment is to ask the question which host proteins may be modulated in terms of expression on viral infection, or indeed across the entire replication cycle. The former has been partially addressed in this study and is also the subject of further manuscripts (3, 36). We found proteins with diverse and potentially relevant functions regulated on SARS-CoV-2 infection in Vero E6 cells but acknowledge that much of the discussion below is of speculative nature at the present time. For instance, the observed expression changes of several transcriptional regulators including MIER1, which regulates the transcription of many SP1 target genes (37), may in part explain the predominantly observed decrease in protein expression on infection. Further, the transcription factor HIF1A, which is thought to act as a bridge between metabolism and innate immunity (38), and other immune-modulatory proteins were strongly down-regulated at high virus dose. This may reflect the fact that Vero E6 cells are permissive for many viruses. Conversely, the up-regulation of other proteins associated with innate immunity (*e.g.* GBP1, MASP1) only at low viral inoculation may reflect the ability of the host cell to mount some antiviral response that, however, is overpowered by other factors at higher virus load. Many of the observed expression changes can be anticipated to remodel the extracellular matrix. For instance, broad regulation of proteases and protease inhibitors were observed as well as enzymes that regulate chondroitin sulfate (CHSY1) and phosphatidylcholine (ABCB4) concentrations. Further, proteins involved in lipid homeostasis (*e.g.* APOE, GRAMD1A) showed expression changes. Up-regulation of the cholesterol transporter GRAMD1A is interesting because this protein is required for the formation of autophagosomes, a key component of the autophagy pathway known to be involved in most viral infections. Clearly, much more work is required to delineate which of the many observed expression changes are cause or consequence during viral infection and host response.

One of the challenges with the interpretation of relatively simple proteomic experiments that just compare protein expression in the presence or absence of virus at a fixed time point is that they do not properly capture the temporal aspects of the infection that makes it difficult to trace causes and consequences. One solution to this challenge is systematic time-resolved experiments (3, 39) and the availability of TMT 6-16-plex reagents provides a means for doing so at a rather high resolution. Related, combining metabolic SILAC-pulsing with TMT labeling at different time points (3, 40, 41, 42) can also be highly informative to monitor the fates of new *versus* old proteins on viral infection over time. Similar arguments can be made to motivate experiments using different titers of inoculating virus. Few such experiments have been performed for SARS-CoV-2 thus far (36, 43, 44). For any of the above mentioned approaches, it would be particularly interesting and potentially important to systematically compare MERS (middle east respiratory syndrome)-CoV, SARS-CoV, and SARS-CoV-2 to improve the understanding of similarities and differences in the cell biology and pathophysiology of these viruses.

Fourth, viral infections are tightly connected to protein-protein interactions (PPI) at almost every step during the replication cycle and performing such studies at a proteomic scale has led to much insight. PPI mapping can be of tremendous value in uncovering cellular processes and it is therefore no surprise that the first MS-based proteomic publication on SARS-CoV-2 indeed presents such an interaction network (2). Some of the proteins identified as potential interactors by Gordon *et al.* in HEK293 cells were also found to be significantly regulated in our study in Vero E6 cells. Even though some of these proteins can be targeted pharmacologically, the effect on virus growth is not clear. One the one hand, one may speculate that some of the respective drugs may be repurposed to target vulnerabilities in biological processes that are vital for the replication of the virus. On the other hand, it is also conceivable that inhibition of these proteins may lead to detrimental effects. Among others, the work of Gordon *et al.* suggests that cAMP-dependent protein kinase PRKACA may be a drug target as it was found to interact with the viral helicase nsp13 and it is also up-regulated at low viral inoculation in our infectome. But the PKA inhibitor H89 had no effect on viral infection. A second example is CYB5R3, a NADH-cytochrome b5 reductase, which is involved in lipid metabolism and upregulated at low virus inoculation. Preclinical inhibitors have been generated for this protein (45), but no information is available on its effect on viral infectivity or production. These compounds, among other effects, have been shown to lead to increased nitric oxide bioavailability and systemically reduce blood pressure in rats, but it is difficult to speculate at this stage if such drugs would have an overall beneficial effect in COVID-19 patients.

Experience shows that integrating several (static) protein interaction networks or measuring PPI network dynamics in response to perturbations can provide deeper insights. Given several such reports on preprint servers, this will likely become possible for SARS-CoV-2 soon (8, 39, 46, 47, 48, 49). Today, MS-based proteomics using tagged proteins, proximity labeling, centrifugation or size-exclusion chromatography-coupled protein correlation profiling are at the technical heart of such investigations and can be powerfully complemented by more recent approaches that localize proteins to subcellular compartments (50, 51, 52, 53, 54). Collectively, the aligned view of multiple proteomic data sets may inform the choice of proteins for follow-up studies aiming at elucidating their role in viral biology, host response or as potential targets for drug discovery.

Fifth, this report and others have shown that SARS-CoV-2 proteins can be efficiently detected by MS and some have even demonstrated this for samples isolated from infected individuals (55, 56, 57, 58, 59). The interpretation of these results, however, vary. Our data on 91 clinical cases shows that the limited dynamic range of direct PRM-MS cannot cope with the proteome complexity of digested nasopharyngeal samples. As a result, a mere 20% of the PCR-positive cases could also be identified by PRM-MS. In addition, the throughput of our PRM-based test is currently limited to 72 samples/day (20 min/sample). In rather stark contrast, a recent study from Brazilian researchers (59) analyzed 562 specimens and reported a 83% confirmation rate of positive PCR results by selected reaction monitoring (SRM) on a triple quadrupole mass spectrometer and a sample throughput of 500 samples per day (2.5 min/sample). This was made possible by employing automated SP3-based sample preparation and an online sample-cleanup method using four parallel turbulent flow chromatography columns coupled to four analytical columns that fed the same mass spectrometer and that monitored three peptides of the NCAP protein. This is an interesting setup as the turbulent flow LC enables the removal of many unwanted compounds (*e.g.* small molecules, peptides) so that the dynamic range and sensitivity of the SRM-MS system can be used more efficiently. The weakness of the approach is that SRM measurements on low-resolution instruments suffer from the inability to verify peptide identity within the experiment because the full tandem mass spectrum is not recorded. Besides, the authors also did not include stable isotope-labeled reference peptides in the analysis, which we found to be essential. An additional challenge for any MS-based approach is to define the criteria for calling a peptide sequence unique to SARS-CoV-2. We chose to gauge our definition to all peptide sequences present in Uniprot and further nonredundant sequences from GenPept, Swissprot, PIR, PDF, PDB, and RefSeq. Other researchers confined their definition to coronaviruses known to be able to infect humans. Neither is right or wrong as it is impossible to ascertain that certain sequences may not exist in other viruses simply because the sequences are not deposited in sequence databases. Nucleotide level approaches have clear advantages here as the degeneracy of the genetic code collapses many codons into the same amino acid, which cannot be distinguished by MS. It could be argued that MS-based proteomics is an untargeted diagnostic method that could play a role in identifying new pathogens or possibly to identify a pathogen at initial presentation of a patient. Although this may be the case in a research setting or in particular clinical circumstances or environments, large-scale population testing will continue to require the sensitivity and scalability of PCR-based detection methods.

## OUTLOOK

Although not covered in this work, it is well established that virus-host interactions are heavily reliant on post-translational modifications (PTMs) on virus and host proteins. Both the virus spike protein as well as the host receptors ACE2 and TMPRSS2 are glycoproteins and several studies are emerging that characterize the glycosylation pattern of individual proteins (60, 61, 62, 63). Unfortunately, global and protein-specific surface glycosylation profiling is still challenging but could become an important future avenue to understand better how complex glycan patterns both of the virus as well as host cells determine the tropism of SARS-CoV-2. A rapidly rising number of reports on SARS-CoV-2 implicate intracellular phosphorylation events in a plethora of pathways from growth factor signaling to interferons and interleukins, HIF1A/mTOR signaling, PIKFYVE kinase inhibition, and JAK1 signaling (39, 64, 65, 66, 67, 68, 69), many of which are implicated in innate immunity. The full extent of phosphorylation regulation (or other PTMs for that matter) by SARS-CoV-2 remains to be elucidated. Because SARS-CoV-2 is an RNA virus, it would also be interesting to investigate how viral RNA interacts with host proteins to trigger virus production. Needless to say, that MS-based proteomics will have a major role to play in these investigations.

Perhaps the most important immediate medical need is to identify existing drugs that can be used to fight COVID-19. This is because the development of vaccines is uncertain, their mass production takes time and herd immunity seems still a long way out at present. At the time of writing, ClinicalTrials.gov lists a staggering 1,270 interventional trials for COVID-19 of which ∼380 are phase III and phase IV trials using existing drugs, procedures, or combinations thereof. Perhaps not surprisingly, many trials are concerned with managing complications observed for many patients such as blood coagulation or severe inflammation. A large group of trials tests antivirals originally developed against other viruses such as Remdesivir (Ebola), Favipiravir, Umifenovir, Oseltamivir (Influenza), Lopinavir, Ritonavir, lopinavir (HIV) and Danoprevir (HCV). Further, there are literally hundreds of trials testing the hypothesis that the anti-inflammatory/anti-malaria drug Hydroxychloroquine (or Chloroquine) may have antiviral activity based on *in vitro* testing. The drug exhibits several activities that are likely the result of polypharmacology including inhibition of toll-like receptors, inhibition of autophagy or aggregation of cytotoxic heme (70, 71, 72). This drug is particularly controversially discussed because, among other factors, results of trials enrolling critically ill patients are difficult to interpret and the drug can cause severe side effects. For presumed desirable or undesirable “off-target” effects, chemical proteomics can have an important role to play, particularly for the identification of the molecular targets as well as their cellular mechanisms of action. Examples for how this may be accomplished are thermal proteome profiling (TPP), limited proteolysis (LiP-MS) or fishing for drug-target interactions using immobilized compounds (73, 74, 75). To the best of our knowledge, no such studies have been reported yet for SARS-CoV-2.

The short-, medium- and long-term clinical management of COVID-19 patients could benefit tremendously from the availability of biomarkers that may be used to monitor or even predict the course of the disease, response to therapy or long-term recovery and prognosis. This is an area of clinical proteomics that holds many promises, but which is also difficult to realize. First steps in the analysis of SARS-CoV-2 patient sera by LC–MS/MS have been taken exemplified by two recent reports that used MS and machine learning to classify Covid-19 patients (4, 5). Although somewhat preliminary given the small number of cases investigated so far, broad or focused proteomic measurements of patient sera may become important sources of information, particularly when performed longitudinally over extended periods of time and when complemented with other technologies such as cytokine arrays that measure the levels of proteins that are difficult to detect by MS. This area of clinical proteomics is still undergoing substantial development with promising progress over the recent past.

In conclusion, it appears quite clear already that MS-based proteomics can make valuable contributions to basic and translational SARS-CoV-2 research. It will be interesting to watch over the coming months if this potential also extends to clinical applications such as diagnostic testing, therapeutic stratification, or recovery monitoring of Covid-19 patients.

## DATA AVAILABILITY

The DDA proteomics raw data, MaxQuant search results and used protein sequence databases have been deposited with the ProteomeXchange Consortium via the PRIDE partner repository and can be accessed using the data set identifier PXD019645. Spectra identifying modified peptides and proteins based on single peptide matches can be visualized from the ‘combined’ folder and the mqpar.xml file from the MaxQuant output using the integrative proteomics data viewer PDV (76). All PRM raw data and Skyline analysis files have been deposited to Panorama Public (https://panoramaweb.org/SARS-CoV-2.url).
