# Supplementary material for: Data, Reagents, Assays and Merits of Proteomics for SARS-CoV-2 Research and Testing
Source: Mol Cell Proteomics. 2020 Nov 25;19(9):1503–22. doi: 10.1074/mcp.RA120.002164 (PMC7780043; doi:10.1074/mcp.RA120.002164)
Supplement: Supplementary file 1 [file mmc1.zip › mmc1/161399_2_supp_555131_qcfxfc.pdf]

Data, reagents, assays and merits of proteomics for SARS-CoV-2 research and testing

Jana Zecha[1*], Chien-Yun Lee[1*], Florian P. Bayer[1], Chen Meng[2], Vincent Grass[3,4], Johannes Zerweck[5], Karsten Schnatbaum[5], Thomas Michler[3], Andreas Pichlmair[3,4] Christina Ludwig[2#], Bernhard Kuster[1,2#]

*equal contribution

# Corresponding authors: tina.ludwig@tum.de, kuster@tum.de

Affiliations

[1] *Chair of Proteomics and Bioanalytics, Technical University of Munich, Freising, Germany*

[2] *Bavarian Center for Biomolecular Mass Spectrometry (BayBioMS), Technical University of Munich, Freising, Germany*

[3] *Institute of Virology, School of Medicine, Technical University of Munich, Munich, Germany*

[4] *German Center for Infection Research (DZIF), Munich partner site, Germany*

[5] *JPT Peptide Technologies, Berlin*

Supplemental Figures and Information

- Supplemental Figures 1-12
- Supplemental Methods
- Supplemental Discussion
- Supplemental References

Supplemental Figures

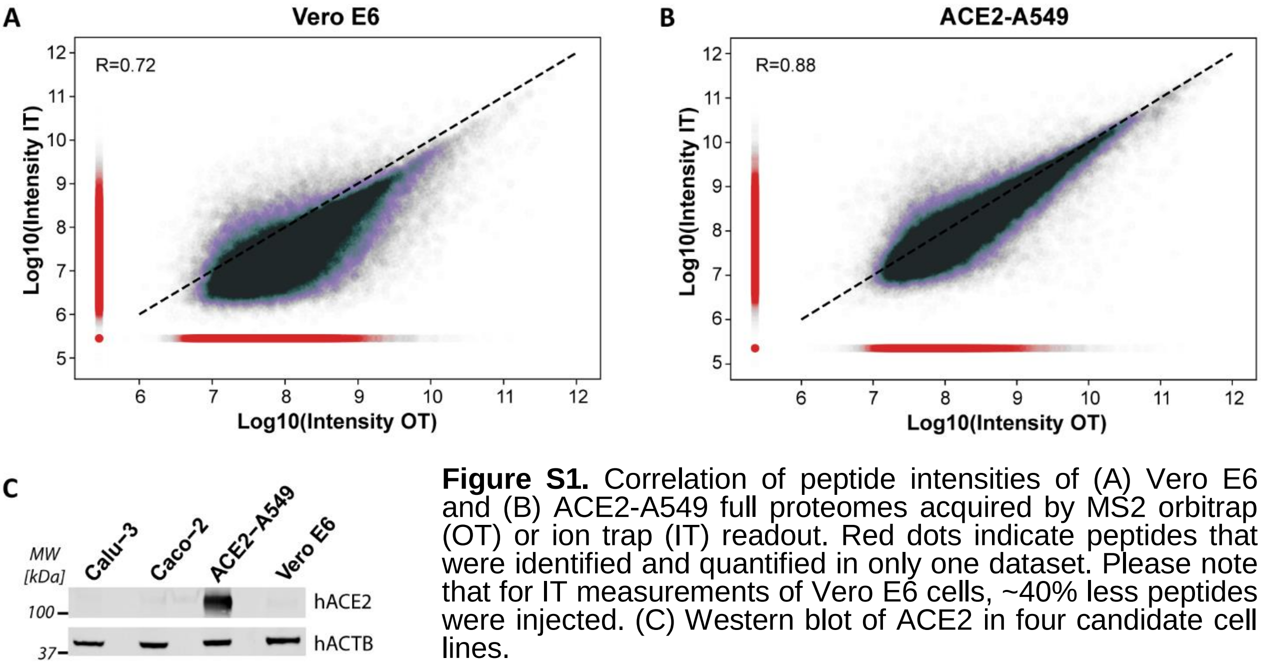

Figure S1. Correlation of peptide intensities of (A) Vero E6 and (B) ACE2-A549 full proteomes acquired by MS2 orbitrap (OT) or ion trap (IT) readout. Red dots indicate peptides that were identified and quantified in only one dataset. Please note that for IT measurements of Vero E6 cells, ~40% less peptides were injected. (C) Western blot of ACE2 in four candidate cell lines.

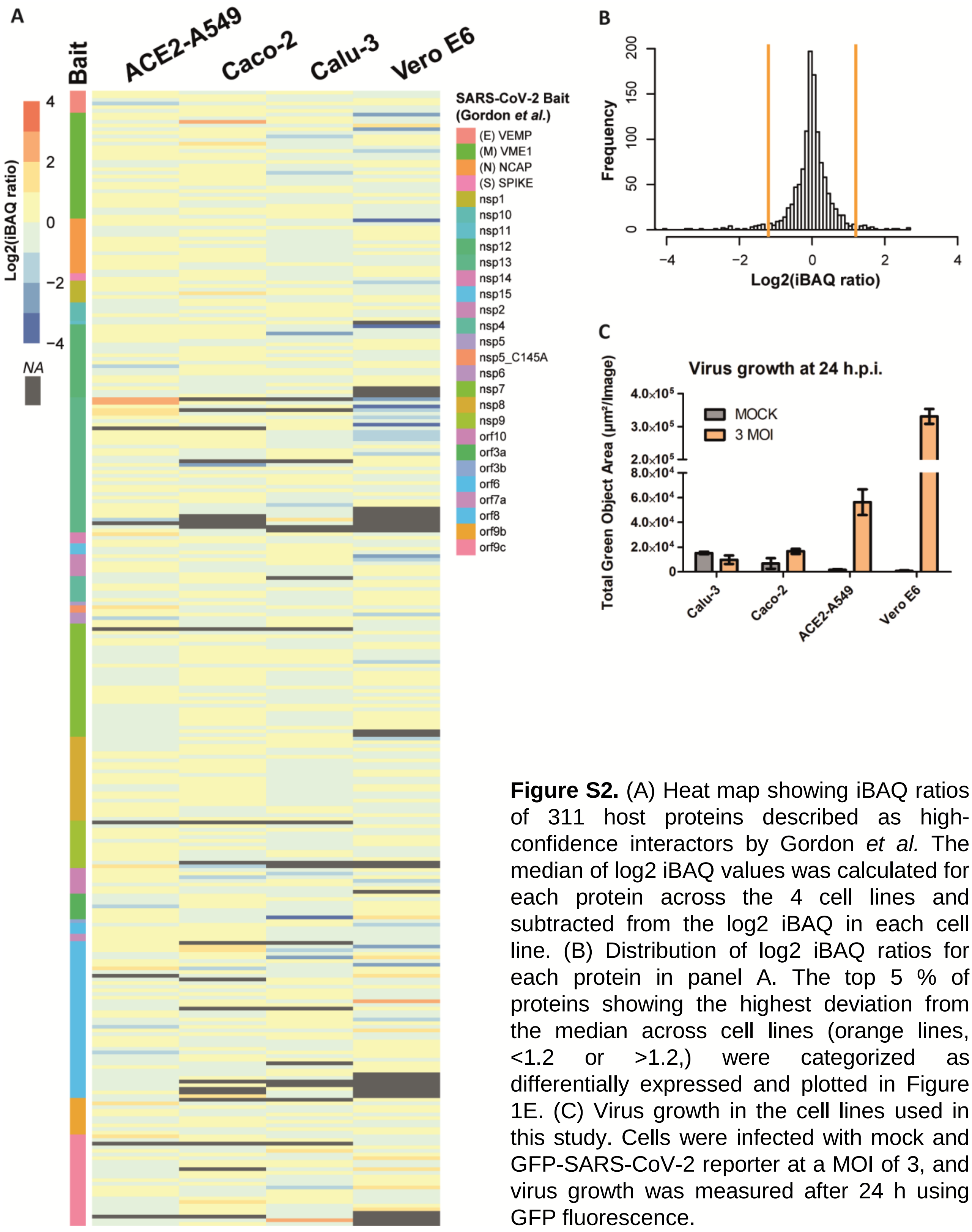

Figure S2. (A) Heat map showing iBAQ ratios of 311 host proteins described as high-confidence interactors by Gordon *et al.* The median of log2 iBAQ values was calculated for each protein across the 4 cell lines and subtracted from the log2 iBAQ in each cell line. (B) Distribution of log2 iBAQ ratios for each protein in panel A. The top 5 % of proteins showing the highest deviation from the median across cell lines (orange lines, <1.2 or >1.2,) were categorized as differentially expressed and plotted in Figure 1E. (C) Virus growth in the cell lines used in this study. Cells were infected with mock and GFP-SARS-CoV-2 reporter at a MOI of 3, and virus growth was measured after 24 h using GFP fluorescence.

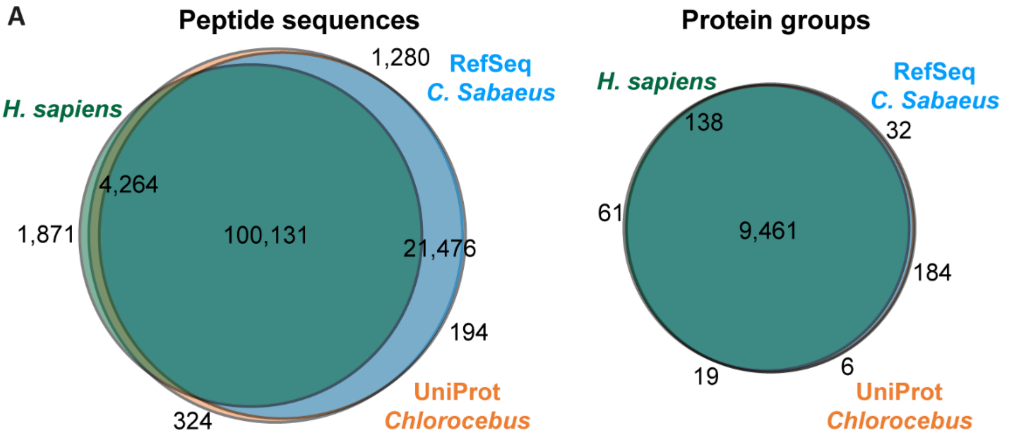

B

# ‚Human-only‘ peptides	# Protein Groups
29	1
11	1
5	4
4	1
3	5
2	4
1	45

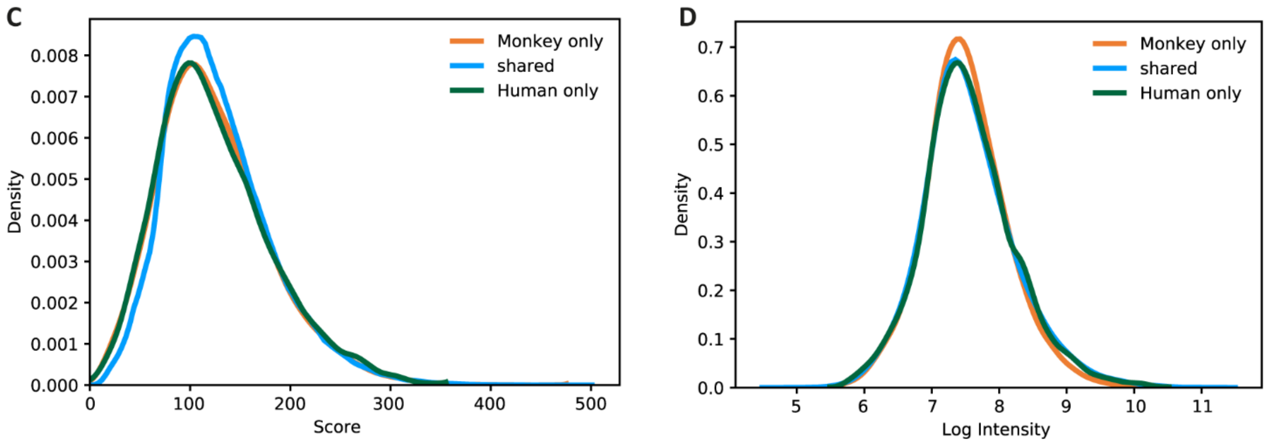

Figure S3. (A) Venn diagrams comparing the peptide and protein group results of a database search of the Vero E6 LC-MS/MS data against human (Uniprot) and *Chlorocebus* (Uniprot and RefSeq) databases. (B) Table summarizing the number of identified peptides for the 61 proteins that could only be identified in the human sequence database. (C) Andromeda score distributions for human-only, monkey-only and shared peptides. (D) The same as (C) but for MS1 intensities.

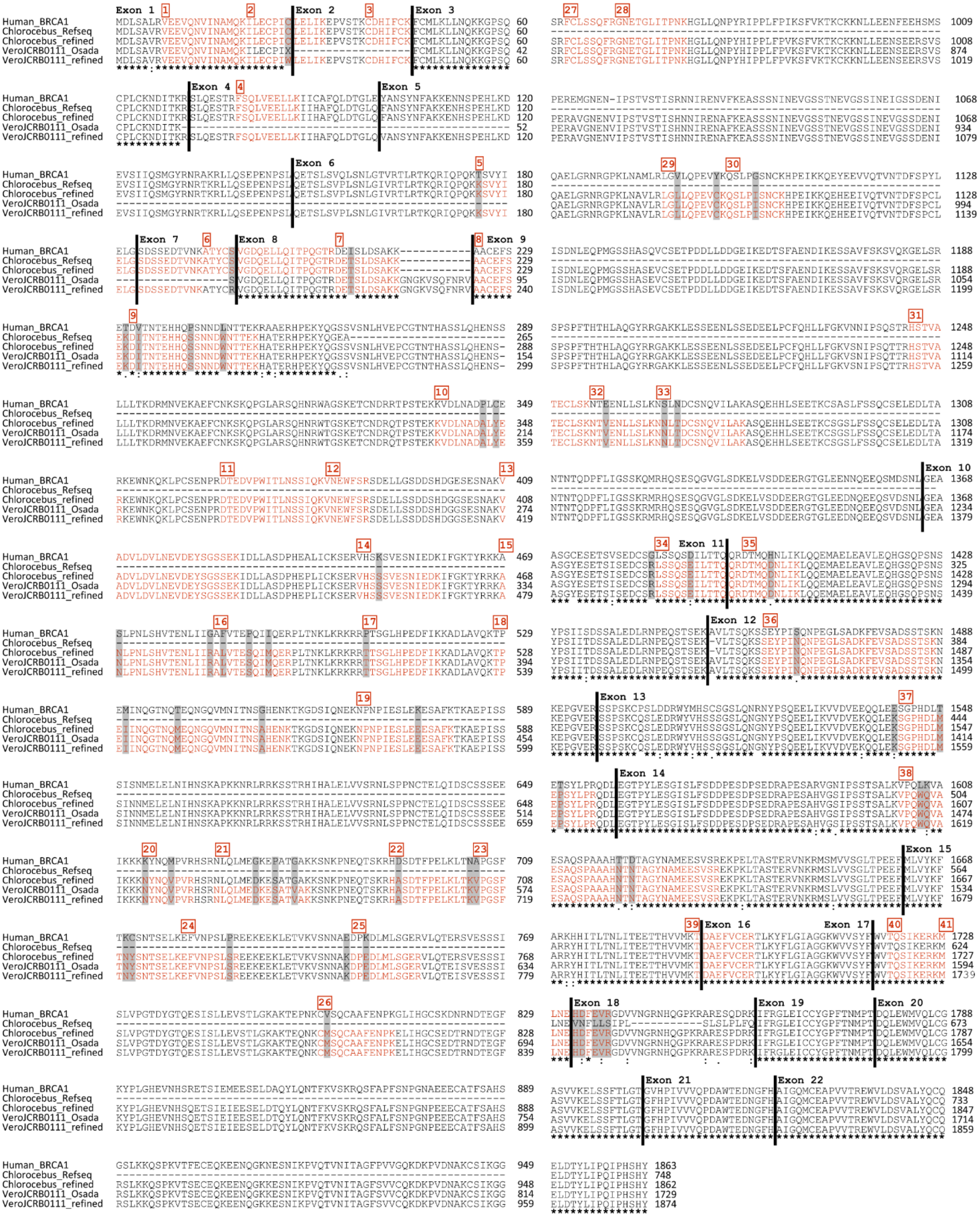

(Fig. continued overleaf)

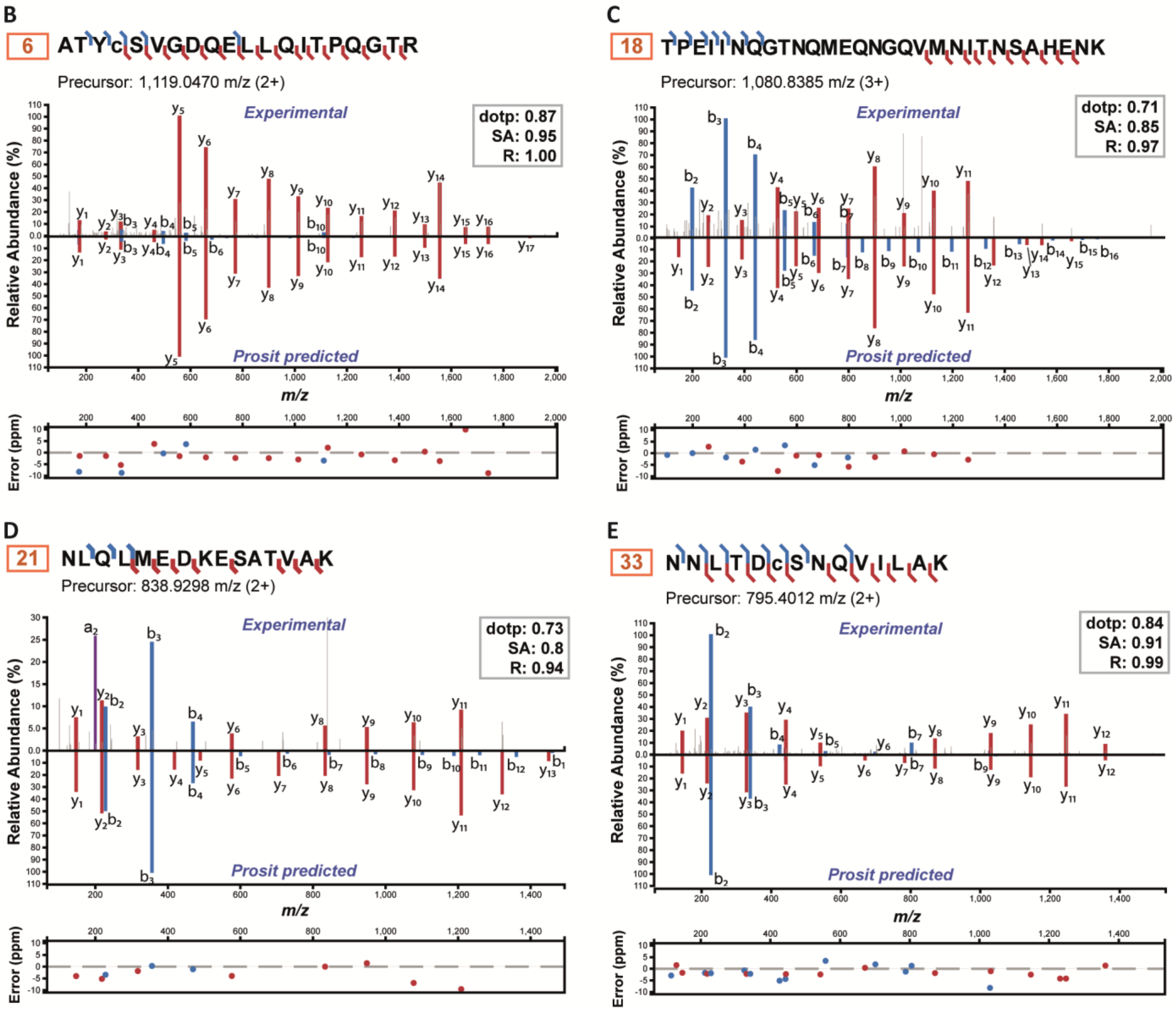

Figure S4. (A) Alignment of the human amino acid sequence for BRCA1 (P38398; Human_BRCA1), the corresponding, annotated *Chlorocebus sabaeus* (Refseq XP_008011633.1; Chlorocebus_Refseq) and Vero JCRB0111 sequences (VeroJCRB0111_Osada), and the refined sequences derived from 6-frame translations of the Refseq *Chlorocebus* and the Vero JCRB0111 DNA sequence assemblies (Chlorocebus_refined, VeroJCRB0111_refined). Peptides that were identified with MaxQuant are indicated in red and numbered consecutively (red boxes). Database searching was performed for 48 high pH reversed phase fractions of the Vero E6 proteome and using either the Refseq *Chlorocebus* and the Uniprot *Chlorocebus* plus the Uniprot human databases or the Refseq *Chlorocebus* database plus the refined protein sequences for BRCA1 and SRP9. SAAV across aligned sequences are marked with grey boxes only within identified peptides. (B-E) Mirror and mass deviation plots of the experimental and Prosit predicted spectra of identified peptides 6 (only annotated in (refined) Refseq), 18 (annotated in refined Refseq and (refined) Vero JCRB0111), 21 (only annotated in (refined) Vero JCRB0111), and 33 (annotated in refined Refseq and (refined) Vero JCRB0111). The similarity of spectra is measured by the dot product (dotp), spectral contrast angle (SA) and Pearson correlation coefficient (R).

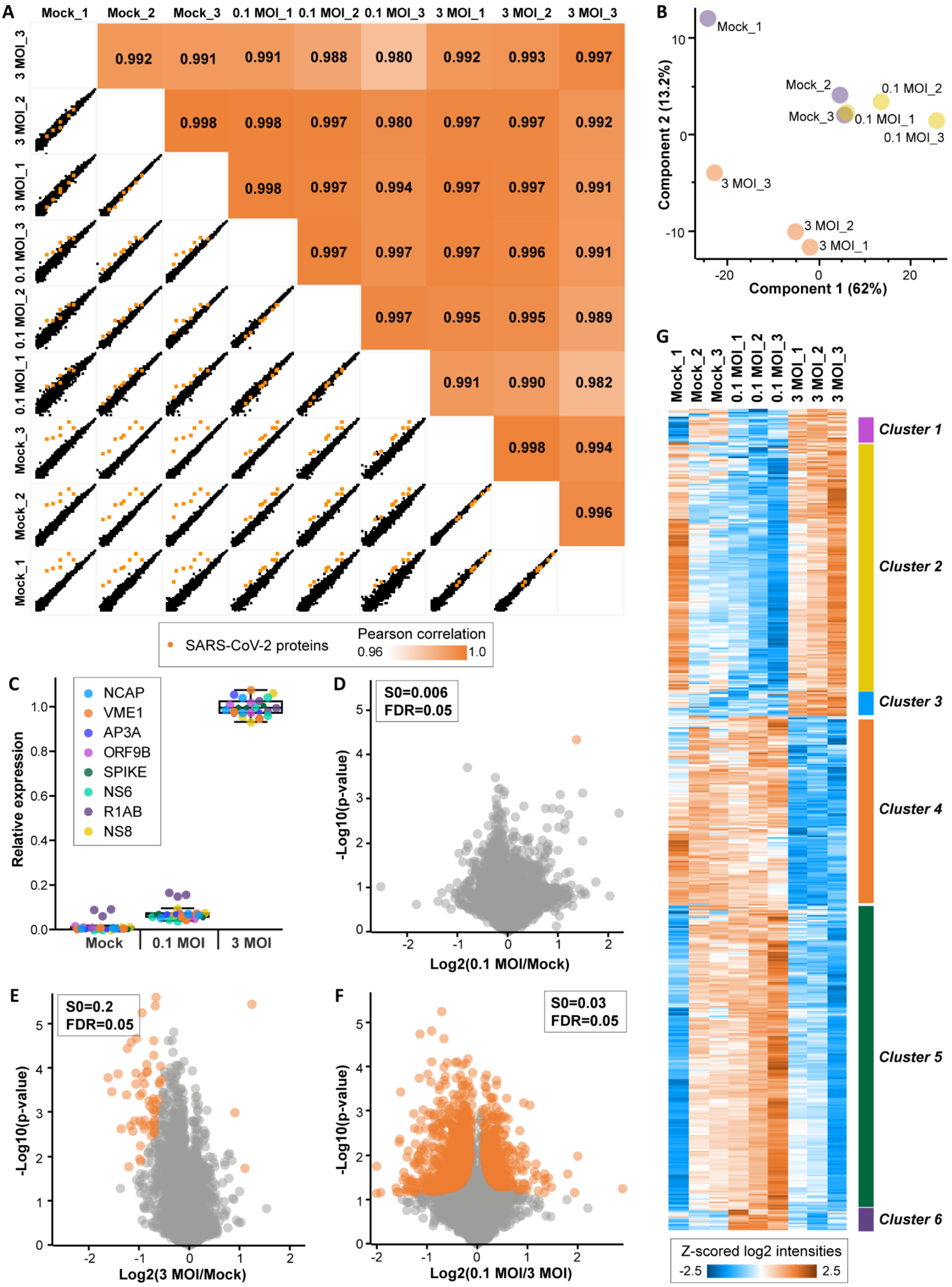
A
Mock_1
Mock_2
Mock_3
0.1 MOI_1
0.1 MOI_2
0.1 MOI_3
3 MOI_1
3 MOI_2
3 MOI_3
0.992 0.991 0.991 0.988 0.980 0.992 0.993 0.997
0.998 0.998 0.997 0.980 0.997 0.997 0.992
0.998 0.997 0.994 0.997 0.997 0.991
0.997 0.997 0.997 0.996 0.991
0.997 0.995 0.995 0.989
0.991 0.990 0.982
0.998 0.994
0.996
SARS-CoV-2 proteins
Pearson correlation
0.96
1.0
B
Component 2 (13.2%)
Component 1 (62%)
C
NCAP
VME1
AP3A
ORF9B
SPIKE
NS6
R1AB
NS8
Relative expression
Mock
0.1 MOI
3 MOI
D
S0=0.006
FDR=0.05
-Log10(p-value)
Log2(0.1 MOI/Mock)
E
S0=0.2
FDR=0.05
Log2(3 MOI/Mock)
F
S0=0.03
FDR=0.05
Log2(0.1 MOI/3 MOI)
G
Cluster 1
Cluster 2
Cluster 3
Cluster 4
Cluster 5
Cluster 6
Z-scored log2 intensities
-2.5
2.5

Figure S5. Further details on the analysis of Vero E6 infectomes after infection with SARS-CoV-2 at 0.1 MOI and 3 MOI (and mock) for 24 h in triplicates. (A) Scatter plots illustrating the correlations of protein abundances across different conditions and replicates. (B) Results of a Principal Component Analysis showing the global similarities and differences of the experiments and replicates. (C) Boxplots displaying the ratios of viral protein expression after infection with the two virus doses. (D-F) Volcano plots depicting data from pairwise, two-sided Student's t-tests assuming equal variance in groups. Significantly regulated proteins are coloured in orange. Applied S0 values are indicated. Results were corrected for multiple testing using a permutation based FDR at 5 %.(G) Heatmap illustrating z-scored expression changes and main clusters within all 1,499 proteins that were identified as significantly regulated in any of the tests shown in panel (D-F).

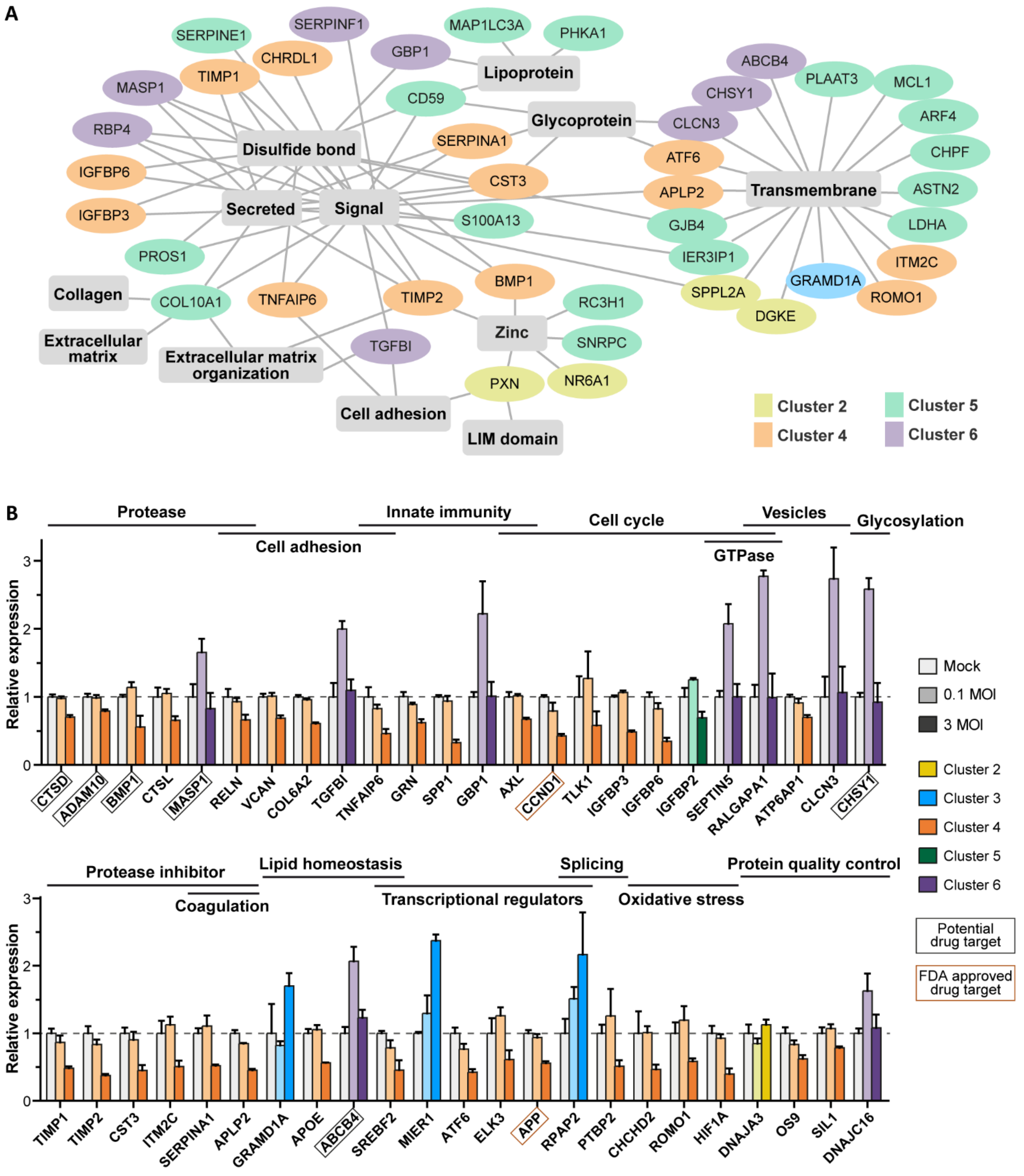

Figure S6. Further details on the analysis of Vero E6 infectomes after infection with SARS-CoV-2 at 0.1 MOI and 3 MOI (and mock) for 24 h in triplicate. (A) Network depicting proteins that are part of enriched functional categories within the 6 main clusters of significantly changing proteins (see Figure 3D) and exhibit at least a 2-fold regulation across any 2 of the 3 conditions. Associations with corresponding functional categories are indicated with lines. (B) Bar charts of regulated proteins from the different clusters for both virus doses.

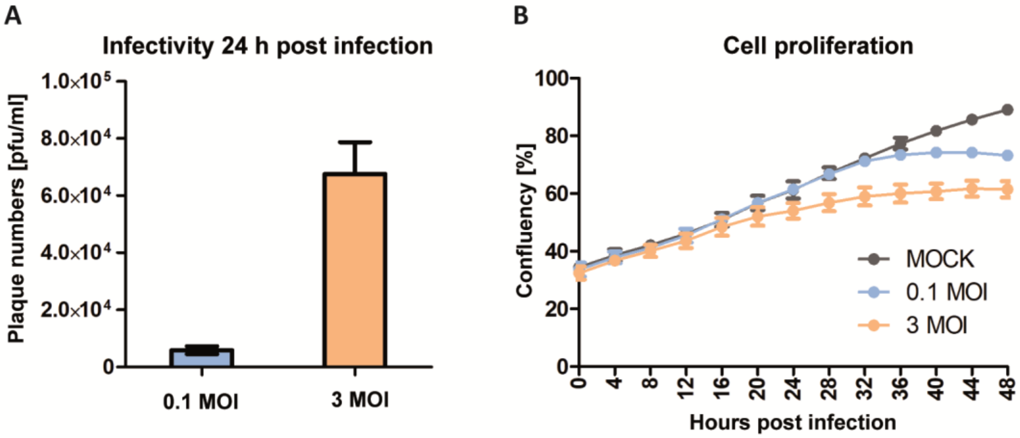

Figure S7. Infectivity and cell proliferation of SARS-CoV-2 infected Vero E6 cells. (A) Plaque assay of Vero E6 cells 24 h after virus infection. (B) Confluence of Vero E6 cells infected with mock, 0.1 MOI and 3 MOI over 48 hours.

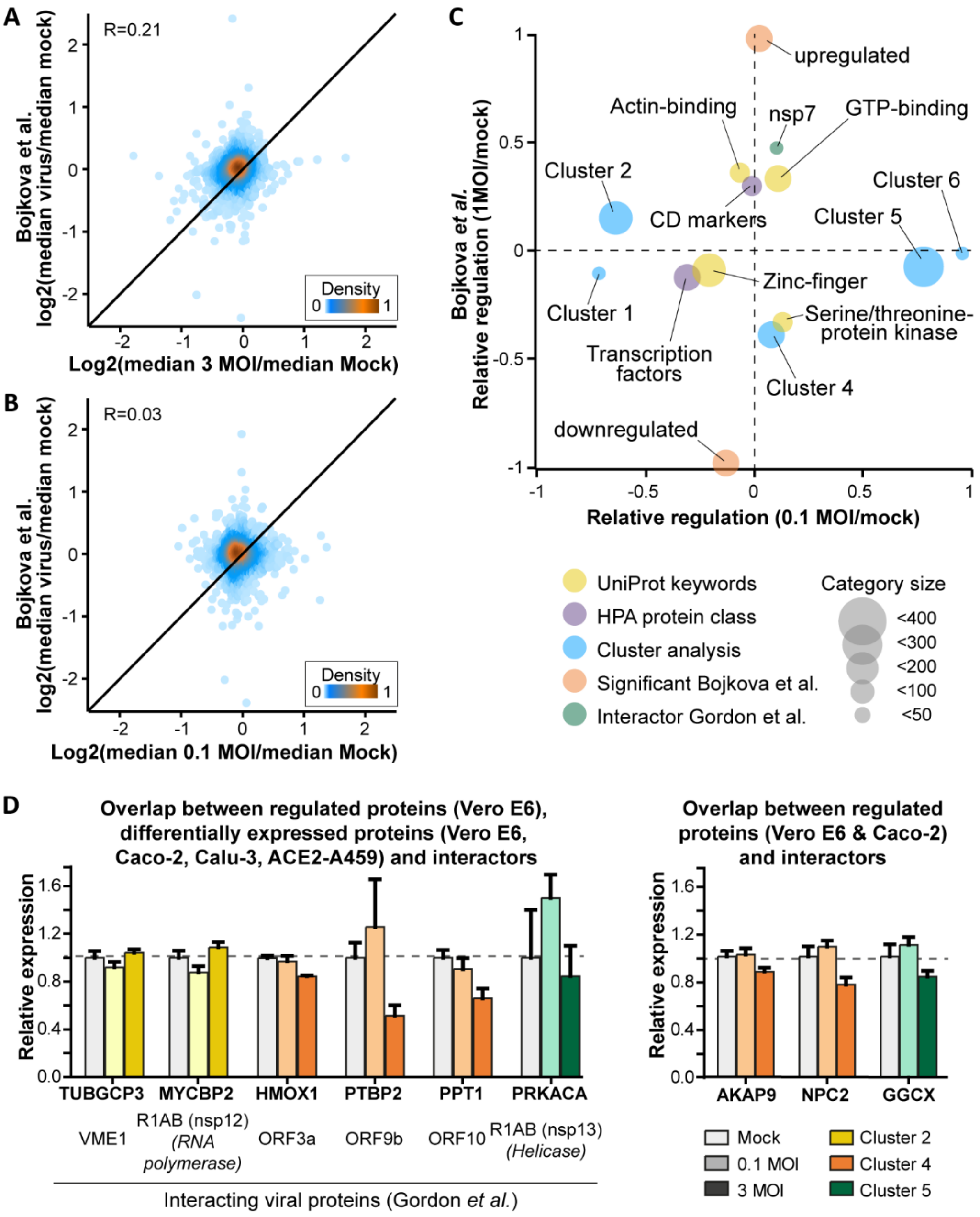

Figure S8. (A) Scatter plot comparing the regulation of host proteins in Vero E6 cells and Caco-2 cells 24 h after infection with SARS-CoV-2 at 3 MOI and 1 MOI, respectively. (B) Same as (A) but after infection of Vero E6 cells at 0.1 MOI. (C) 2D enrichment analysis showing annotation terms whose members show consistent behaviour, i.e. up-regulation (relative regulation>0) or down-regulation (relative regulation<0), in one or both of the virus-host response studies (24 h p.i., Vero E6: 0.1 MOI, Caco-2: 1 MOI). Only categories with a |relative regulation| > 0.2 in at least one of the two datasets are displayed. (D) Bar charts on the left depict regulations of proteins that were identified as significantly regulated in Vero E6 cells after SARS-CoV-2 infection, were categorized as differentially expressed across the 4 candidate cell lines (see Figure S2B), and, additionally, were reported as confident interactors of SARS-CoV-2 proteins by Gordon *et al.*. Bar charts on the right illustrate regulations of the 3 proteins that were identified as significantly regulated in both, our virus-host response study and the one of Bojkova *et al.*, and, additionally, were reported as high-confidence interactors of SARS-CoV-2 proteins by Gordon *et al.*.

A

Proteins	In gel 1	In gel 2	In solution 1	In solution 2	In solution 3	SP3 1	SP3 2	SP3 3
Protein 9b	1	0	4	3	3	0	0	0
Spike glyco-protein	11	7	2	3	2	10	9	7
Membrane protein	4	4	1	1	1	3	3	3
Non-structural protein 8	0	0	1	1	2	1	2	1
Nucleoprotein	14	15	12	11	11	14	17	17
∑ peptides	30	26	20	19	19	28	31	28

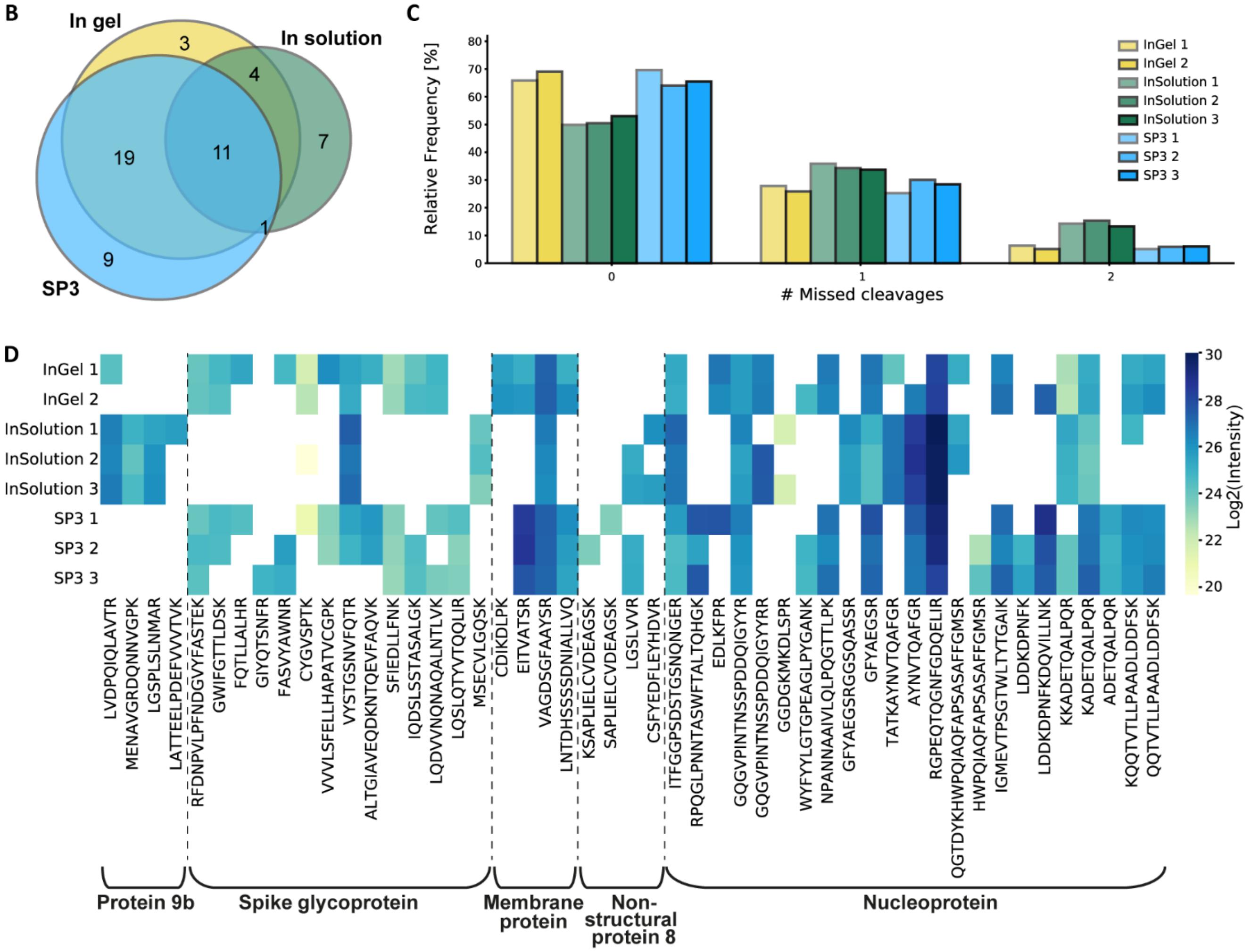

Figure S9. Comparison of different sample preparation workflows for proteomic SARS-CoV-2 analysis. (A) Table displaying the number of tryptic peptides identified per viral protein and workflow. (B) Venn diagram showing the overlap of identified viral peptides between different workflows. (C) Bar charts comparing the rate of tryptic missed cleavages in the different workflows. (D) Heat map visualizing peptide intensities of viral proteins. Missing values are indicated by a white box.

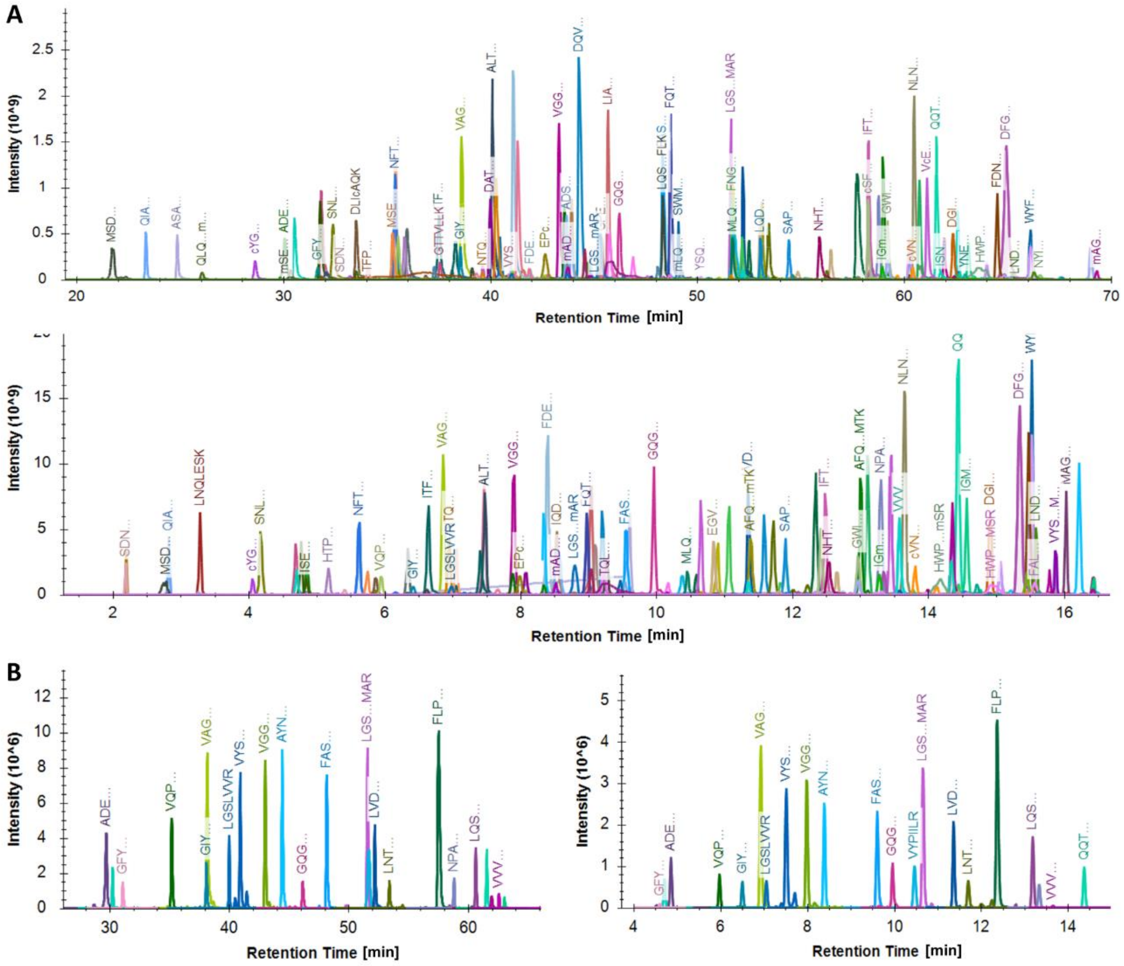

Figure S10. (A) Chromatograms showing the retention times of 98 heavy spike-in peptides from the SARS-CoV-2 peptide library on a nano-flow system (50 min gradient, upper panel) and a micro-flow system (15 min gradient, lower panel). (B) Chromatograms illustrating the retention times of 23 and 21 heavy spike-in peptides from the PRM assay panel for SARS-CoV-2 detection on a nano-flow system (60 min gradient, left panel) and a micro-flow system (15 min gradient, right panel), respectively.

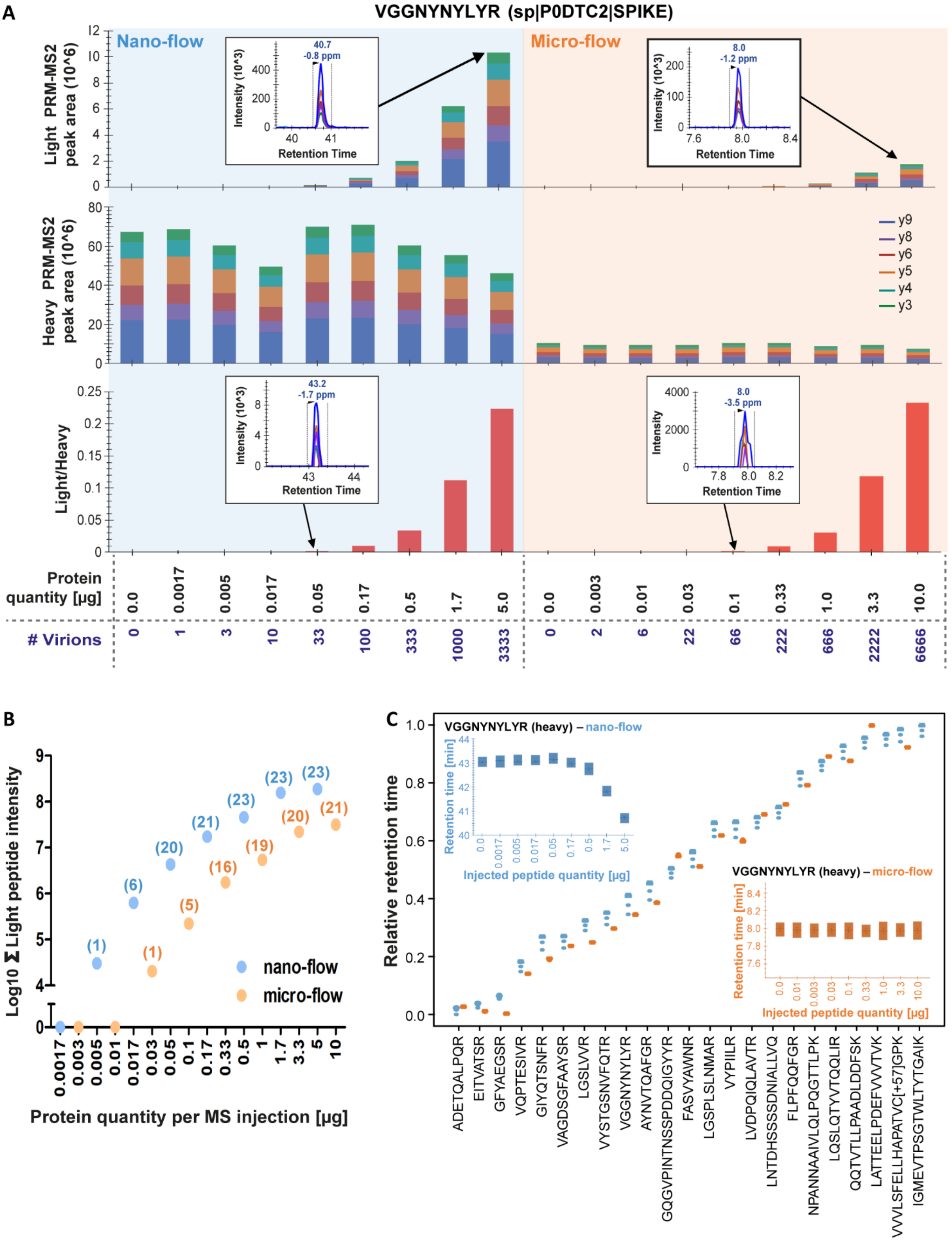
A
VGGNYNYLYR (sp|P0DTC2|SPIKE)
Nano-flow
Micro-flow
Light PRM-MS2 peak area (10^6)
Heavy PRM-MS2 peak area (10^6)
Light/Heavy
Intensity (10^3)
Intensity
Retention Time
40.7
-0.8 ppm
8.0
-1.2 ppm
43.2
-1.7 ppm
8.0
-3.5 ppm
y9
y8
y6
y5
y4
y3
Protein quantity [µg]
0.0
0.0017
0.005
0.017
0.05
0.17
0.5
1.7
5.0
0.0
0.003
0.01
0.03
0.1
0.33
1.0
3.3
10.0
Virions
0
1
3
10
33
100
333
1000
3333
0
2
6
22
66
222
666
2222
6666
B
Log10 Σ Light peptide intensity
nano-flow
micro-flow
Protein quantity per MS injection [µg]
C
Relative retention time
VGGNYNYLYR (heavy) – nano-flow
Retention time [min]
Injected peptide quantity [µg]
VGGNYNYLYR (heavy) – micro-flow
ADETQALPQR
EITVATSR
GFYAEGSR
VQPTESIVR
GIYQTSNFR
VAGDSGFAAYSR
LGSLVVR
VYSTGSNVFQTR
VGGNYNYLYR
AYNVTQAFGR
GQGVPINTNSSPDDQIGYYR
FASVYAWNR
LGSPLSLNMAR
VYPIILR
LVDPQIQLAVTR
LNTDHSSSSDNIALLVQ
FLPFQQFGR
NPANNAAIVLQLPQGTTLPK
LQSLQTYVTQQLIR
QQTVTLLPAADLDDFSK
LATTEELPDEFVVVTVK
VVVLSFELLHAPATVC[+57]GPK
IGMEVTPSGTWLTYTGAIK

Figure S11. (A) PRM results of a dilution series of digested supernatant samples of SARS-CoV-2-infected Vero E6 cells analyzed by nano- and micro-flow PRM-MS. The panels show PRM-MS2 intensities for the 6 most intense fragment ions of a SARS-CoV-2 unique peptide representing the Spike glycoprotein. The intensity of the endogenous (light) peptide is shown in the upper panel, the heavy reference peptide in the middle panel and the ratio of the two in the lower panel. (B) Summed PRM-MS2 signal intensities of all detected SARS-CoV-2 peptides as a function of dilution steps. The number of confidently detected peptides at each dilution step is given in brackets. (C) Retention time variance is depicted for 23 and 21 SARS-CoV-2 heavy spike-in peptides over 9 dilution steps of in-gel digested supernatants of virus producing Vero E6 cells using nano-flow and micro-flow chromatography, respectively. In order to make the nano- and micro-flow data directly comparable, all retention times were normalized to the range from 0 to 1. The two insets show absolute retention times for one isotopically labeled spike-in peptide. In the nano-flow measurements, retention time shifts of up to 1.5 minutes were observed, especially for the highly concentrated sample.

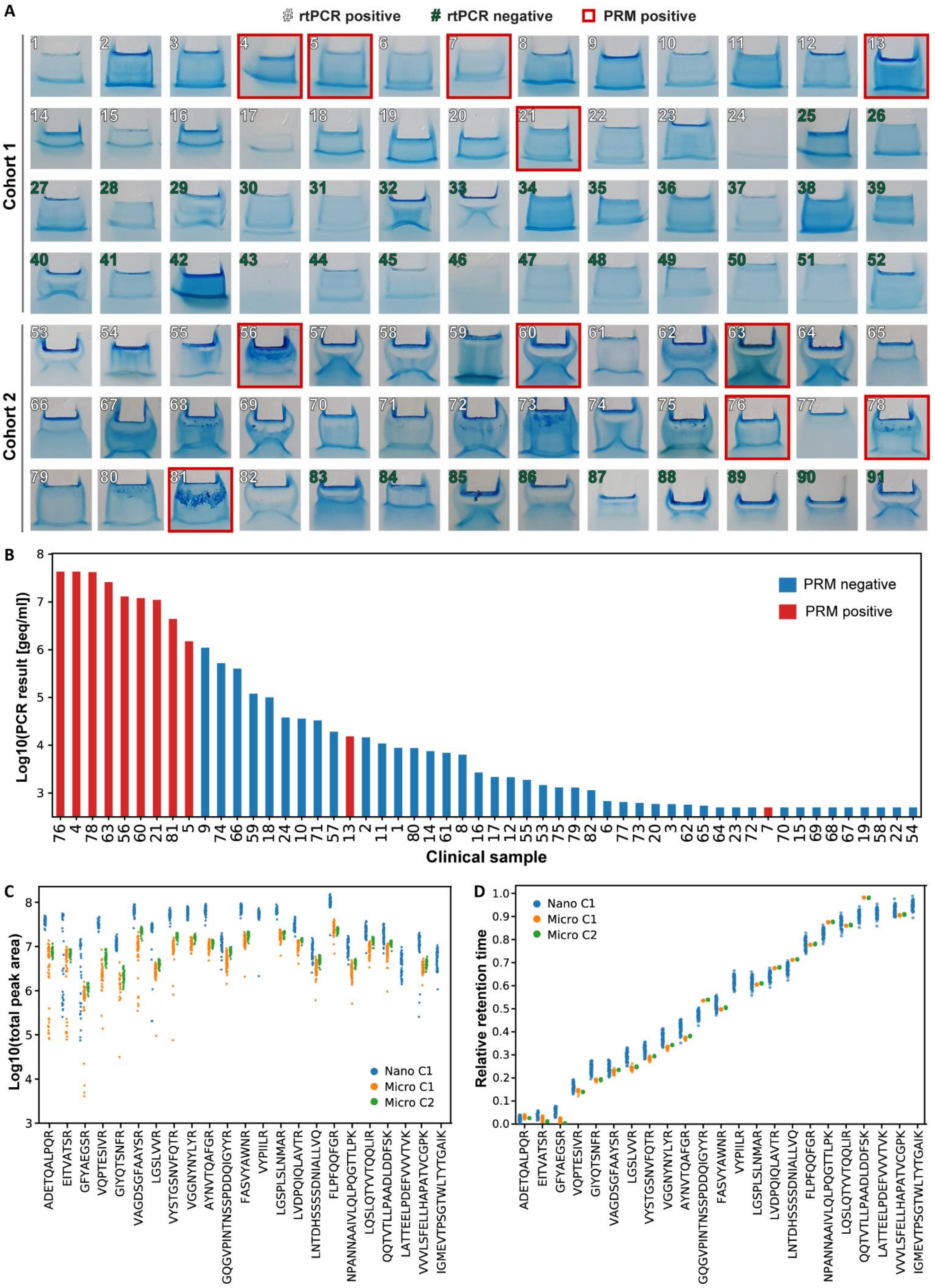
A
rtPCR positive
rtPCR negative
PRM positive
Cohort 1
Cohort 2
B
PRM negative
PRM positive
Log10(PCR result [geq/ml])
Clinical sample
C
Log10(total peak area)
Nano C1
Micro C1
Micro C2
D
Relative retention time
ADETQALPQR
EITVATSR
GFYAEGSR
VQPTESIVR
GIYQTSNFR
VAGDSGFAAYSR
LGSLVVR
VYSTGSNVFQTR
VGGNYNYLYR
AYNVTQAFGR
GQGVPINTNSSPDDQIGYYR
FASVYAWNR
VYPIILR
LGSPLSLNMAR
LVDPQIQLAVTR
LNTDHSSSDNIALLVQ
FLPFQQFGR
NPANNAAIVLQLPQGTTLPK
LQSLQTYVTQQLIR
QQTVTLLPAADLDDFSK
LATTEELPDEFVVVTVK
VVVLSFELLHAPATVCGPK
IGMEVTPSGTWLTYTGAIK

Figure S12. SARS-CoV-2 detection in clinical diagnostic samples by PRM-MS. (A) Gel pictures of 91 diagnostic samples included in this study. Note that for cohort 2 (C2), roughly 13x more material was loaded per gel pocket. (B) PCR-positive samples are ranked by virus load determined by PCR. Red colour indicates PRM-positive, blue indicate PRM-negative cases. (C) Peak areas plotted for each heavy spiked-in peptide. Every dot represents one clinical sample. (D) Same as (C) but for relative retention times of heavy spiked-in peptides.

Supplemental Methods

Samples, reagents, and cell culture assays

Synthetic, isotopically labeled SARS-CoV-2 peptides – In total, 113 isotopically labeled SpikeTides^{TM} peptides covering 11 SARS-CoV-2 proteins were kindly provided by JPT Peptide Technologies (Supplemental Table S3). In these peptides, either the C-terminal lysine (Lys8) or arginine residue (Arg10) was ^{13}C- and ^{15}N-labeled. For peptides located at the C-terminus of a protein that do not entail a C-terminal lysine or arginine residue, leucines were labeled with ^{13}C and ^{15}N (Leu7). All quantities per spike-in peptide specified represent only rough estimates, as the isotopically labelled peptides were not purified and concentrations were not determined accurately.

Cell culture – For generation of an ACE2 expressing A549 cell line (ACE2-A549), C-terminally hemagglutinin-tagged ACE2 was amplified from an ACE2 expression vector (kindly provided by Stefan Pöhlmann (1)) and cloned into the lentiviral vector pWPI-puro. A549 cells were lentivirally transduced, and stably transduced cells were selected with puromycin. ACE2-A549 and Calu-3 cells were cultured in IMDM medium containing 10 % and 20 % FBS (Gibco), respectively. Caco-2 cells were grown in DMEM medium supplemented with 10 % FBS, 1x non-essential amino acids (PAN-Biotech), 1 mM sodium pyruvate (Gibco) and 1 % antibiotic, antimycotic solution (Sigma). Vero E6 cells were cultured in DMEM complemented with 10 % FBS, 100 µg/ml Streptomycin and 100 IU/ml Penicillin.

Virus growth assay – Calu-3, Caco-2, ACE2-A549 and Vero E6 cells were seeded in 12-well plates and infected with SARS-CoV-2 with GFP conjugated (2). An IncuCyte S3 Live-Cell Analysis System was utilized to capture 9 real-time images per well of mock (phase channel) and infected cells (GFP and phase channel) every 24 h for 48 h. Virus growth (mock and infected cells) was assessed as total GFP area. Analysis was performed using the IncuCyte S3 Software (Essen Bioscience).

Plaque assay – Titers of viral isolate and viral stock were determined by plaque assay. Confluent monolayers of Vero E6 cells were infected with serial five-fold dilutions of virus supernatants for 1 h at 37 °C. The inoculum was removed and replaced with serum-free MEM (Gibco) containing 0.5 % carboxymethylcellulose (Sigma-Aldrich). Two days post-infection, cells were fixed for 20 min at room temperature with formaldehyde directly added to the medium to a final concentration of 5 %. Fixed cells were washed extensively with PBS before staining with H_2O containing 1 % crystal violet and 10 % ethanol for 20 min. After rinsing with PBS, the number of plaques was counted and the virus titer was calculated.

Viability assay – Cell viability of Vero E6 cells was obtained as described above but infected with the WT strain SARS-CoV2-MUN-IMB1. The whole well real-time images per well of mock and infected (Phase channel) cells were obtained every 4h for 48h. The confluence was generated using IncuCyte S3 Software (Essen Bioscience).

Collection of respiratory specimens – In this study, only specimens that were collected as part of the standard diagnostic testing and that would normally be discarded were used.

Approval to do so was granted by the ethics committee of the University Hospital "rechts der Isar" of the Technical University of Munich. Personal identification was not recorded, and only SARS-CoV-2 proteins were investigated. For nasopharyngeal swabs, a polyurethane swab (Sigma-swab, Medical wire) was inserted through the nasal canal to the nasopharynx, and specimen was dissolved in 1 ml fluid amies media (Sigma-Transwab®). Bronchoalveolar lavage (BAL) of the lower respiratory tract was performed using a fiber-optic bronchoscope and flushing with 0.9 % saline. Samples were stored at -80 °C until further analysis. Inactivation was performed by heating at 95 °C for 10 min.

Quantification of viral load using Reverse Transcription Polymerase Chain Reaction – Nucleic acid was extracted from 650 µl sample material using the Abbott mSample Preparation System and m2200sp liquid handler (Abbott Laboratories). Elution was performed in 70 µl H_2O. Reverse transcription polymerase chain reaction (RT-PCR) was performed on an Applied Biosystems® 7500 Real-time PCR system (Thermo Fisher Scientific) using 5 µl of the eluate (~4.6 % of the initial sample) and CDC N1 primers (2019-nCoV_N1-F: 5'-GAC CCC AAA ATC AGC GAA AT-3'; 2019-nCoV_N1-R: 5'-TCT GGT TAC TGC CAG TTG AAT CTG-3') and probe (2019-nCoV_N1-P: 5'-FAM-ACC CCG CAT TAC GTT TGG TGG ACC-BHQ1-3') and following cycling conditions: 2 min at 50 °C, 10 min at 95 °C followed by 45× (15 s at 95 °C, 1 min at 60 °C). Quantification was performed using a plasmid standard and lower limit of quantification (LLOQ) of 500 genome equivalents (geq)/ml.

Proteomics sample preparation

SP3 protein digestion and clean up – To hydrolyze DNA and reduce viscosity, cell lysates were heated at 95 °C for 5 min and TFA was added to a final concentration of 1 % (3). Quenching was performed using 3 M Tris, pH 10 (final concentration of ~195 mM, pH 7.8). Protein concentration was determined using the Pierce BCA Protein Assay Kit (Thermo Scientific). The lysate was cleaned up using the SP3 method on an automated Bravo liquid handling system (Agilent) as previously described (4) with minor modifications. Twenty µl of a 1:1 slurry of two types of carboxylate beads (50 µg/µl in H_2O, Sera-Mag Speed beads, cat# 45152105050250 and 65152105050250, GE Healthcare) were added to a 96-well plate (cat#951020401, Eppendorf). After removal of water from bead aliquots placed on a magnet rack (G5498B#008 Magnetic Bead Accessory, Agilent), 200 µg of protein (120 µg for Vero E6 measured with IT MS method) in a total volume of 40 µl lysis buffer (2 % SDS, 40 mM Tris-HCl pH 7.6) were added. Following shaking at 1,000 rpm for 1 min, 100 % acetonitrile (ACN) were added to reach a final concentration of 60 % ACN. The plate was shaken at 1,200 rpm for 10 min and then transferred to the magnet rack for 1 min to immobilize the beads. After removal of the supernatant, the beads were washed twice with 200 µl of 80 % ethanol and once with 180 µl of 100 % ACN. Beads were then reconstituted in 80 µl of digestion buffer containing 50 mM Hepes (pH 8.5), 10 mM Tris(2-carboxyethyl)phosphine (TCEP), and 50 mM chloroacetamide (CAA). Reduction and alkylation was conducted for 1 h at 1,200 rpm and 37 °C, followed by digestion overnight at 37 °C and 1,200 rpm using a 1:50 trypsin-to-protein ratio. The supernatant containing peptides was transferred to a new 96-well plate and acidified by neat formic acid (FA) to reach a final concentration of 1 %. Peptides were desalted using

RP-S cartridges (5 µL bed volume, Agilent) and the standard peptide cleanup v2.0 protocol on the AssayMAP Bravo Platform (Agilent). Briefly, RP-S cartridges were primed with 100 µL of 50% ACN, 0.1% FA and equilibrated with 50 µL of 0.1% FA at a flow rate of 10 µL/min. The samples were loaded at 5 µL/min, followed by an internal cartridge wash with 0.1% FA at a flow rate of 10 µL/min. Peptides were eluted with 80 µL 70% ACN, 0.1% FA at a flow rate of 5 µL/min.

TMT labeling and peptide fractionation of cell line samples – Triplicates of SARS-CoV-2 infected Vero E6 cells were labeled with 9 channels of TMT10plex reagent kit (Thermo Scientific, channel 127N was omitted) according to our previously published protocol (5) with minor modifications. In brief, 100 µg of TMT reagent in 5 µl of anhydrous ACN was used to label 30 µg of peptides in 20 µl of 50 mM Hepes buffer (pH 8.5) and labeling reaction was stopped by adding 3 µl of 5 % hydroxylamine. After vacuum drying, peptides were dissolved in 0.1 % FA and desalted by the AssayMAP Bravo Platform (Agilent) as described above. For off-line high pH reversed phase (RP) fractionation of label-free and TMT-labelled cell lines, a Dionex Ultra 3000 HPLC system equipped with a Waters XBridge BEH130 C18 column (3.5 µm 2.1 × 150 mm) was operated at a flow rate of 200 µl/min with a constant 10 % of 25 mM ammonium bicarbonate (pH = 8.0) in the running solvents. Non-labeled peptides (200 µg) were separated using a 57 min linear gradient from 4 to 32 % ACN in ddH_2O followed by a 3 min linear gradient up to 85 % ACN. For TMT-labeled peptides, a 57 min linear gradient from 7 to 45 % ACN in ddH_2O followed by a 6 min linear gradient up to 80 % ACN was employed. Forty-eight fractions were collected every half minute from minute 3 to 51 and pooled discontinuously into 48 fractions (fraction 1 + 49, fraction 2 + 50, and so on). Peptide fractions were frozen at -80 °C and dried by vacuum centrifugation.

Comparison of digestion protocols for SARS-CoV-2 peptide detection – Supernatant of SARS-CoV-2 infected Vero E6 cells, which contained 2e6 virions (infectious virus particles) per ml as measured by plaque assay, was utilized to evaluate SP3-based, in-gel and in-solution digestion in urea buffer for the detection of SARS-CoV-2 derived peptides. The total protein concentration of the supernatant sample was determined using the Pierce Coomassie (Bradford) Protein Assay Kit (Thermo Scientific). The SP3 workflow was performed as described above with a protein input of 50 µg. For in-solution digestion, 8 M urea lysis buffer (in 40 mM Tris-HCl, pH 7.6 containing protease inhibitors) was added to 50 µg protein to reach a final concentration of 6 M urea. After reduction (10 mM DTT, 30 °C, 30 min) and alkylation (50 mM CAA for 30 min (HeLa), lysates were diluted to 1.6 M urea using 40 mM Tris-HCl, pH 7.6. Digestion was performed by adding trypsin at a 1:50 enzyme-to-substrate ratio and incubating overnight at 37 °C and 600 rpm. Digests were acidified by addition of neat FA to 1 %, centrifuged to pellet insoluble matter, and desalted using tC18 RP solid-phase extraction cartridges (Waters Corp.; wash solvent: 0.1 % FA; elution solvent: 0.1 % FA in 50 % ACN). For in-gel digestion, 16 µg of the cell culture supernatant was mixed 1:1 with 4x Novex NuPage LDS sample buffer (Invitrogen) containing 20 mM DTT. The sample was run 1 cm into a 4-12 % Bis-Tris-protein gel using 1x MOPS SDS running buffer (Novex NuPage, Invitrogen). Reduction (10 mM DTT in 5 mM TEAB, 45 min), alkylation (50 mM CAA in 5 mM TEAB, 30 min), and overnight digestion of proteins (250 ng trypsin in 5 mM TEAB) was performed

according to standard in-gel procedures. Cleaned-up peptides from all samples were frozen at -80 °C, vacuum dried and stored at -20 °C until measurement.

Selection of ACE2, TMPRSS2, and SARS-CoV-2 target peptides and PRM assay development

PRM assays were designed in accordance with the Tier 3 guidelines for targeted assay development (6, 7). Tier 3 measurements are PRM assays that enable repeatable measurements of the same sets of analytes across large-scale experiments, but do not aim for accurate and precise quantification. Additionally, we used isotopically labeled internal reference peptide for all our targeted SARS-CoV-2 peptides for maximally confident peptide identification in clinical samples.

Generation of a peptide library for human and monkey ACE2 and TMPRSS2 – Selection of target peptides for ACE2 and TMPRSS2 were based on results of DDA measurements of deep cell line proteomes. For human ACE2, the 15 most intense peptides were picked in their respective most intense charge state. For human TMPRSS2, all of the six peptides detected by DDA were included for the PRM measurement. For monkey ACE2, the eight peptides identical to targeted human ACE2 peptides were selected. Additionally, one monkey-specific peptide was added, which was detected in the Vero E6 cell line in DDA mode. For monkey TMPRSS2, no peptides were detected in Vero E6 by DDA. Thus, a TMPRSS2 peptide shared between human and monkey was included since this peptide has been detected in the human samples. Further, five monkey-specific TMPRSS2 peptides were selected, which corresponded to detected human peptides, but carried single amino acid variants (in part leading to new cleavage sites). Those were included in two charge states to increase the probability of detection. In total, 21 and 15 peptide sequences were targeted for the three human and one monkey cell line, respectively (Supplemental Table S1). Spectral libraries were built from experimental spectra of deep proteome measurements and predicted spectra using Skyline (version 20.1.1.83, (10)) and the Prosit 2019 algorithm (11). All data are available for download from Panorama Public (12) (https://panoramaweb.org/SARS-CoV-2.url).

Generation of a peptide library for SARS-CoV-2 – Targeted SARS-CoV-2 peptide selection started with the *in silico* tryptic digestion of the Uniprot derived SARS-CoV-2 proteome (UP000464024, 14 entries, last modified on 22nd of March 2020). All tryptic peptides with a length constraint between 7 to 24 amino acids were selected. The Replicase polyproteins 1ab and 1a were excluded, as we reasoned that those proteins, which do not make up the actual viral particle, will be of rather low abundance in samples taken from COVID-19 suspected individuals. The non-structural 7b protein (NS7B) did not lead to a single peptide shorter than 24 amino acids and could therefore not be targeted by PRM. In total, 113 peptides representing 11 proteins met our selection criteria. All peptides were synthesized as SpikeTides^{TM} in isotopically labeled form (JPT Peptide Technologies) and pooled into a single peptide mix. Nano-flow PRM measurements of this mix showed confident detection of 98 out

of the 113 peptides (MaxQuant score > 50, Supplemental Table S3). Skyline (version 20.1.1.83, (8)) was used to build experimental spectral libraries from generated DDA as well as PRM data processed with MaxQuant (version 1.6.3.4 (9)). Additionally, we also predicted a spectral library using the Prosit 2019 algorithm (10). All libraries entail high-quality reference spectra and retention time information for the 98 detected SARS-CoV-2 peptides and are available for download from Panorama Public (11) (https://panoramaweb.org/SARS-CoV-2.url). Micro-flow LC-MS/MS measurements of the heavy SARS-CoV-2 peptide mix identified 96 of the 98 synthetic peptides with high confidence (MaxQuant score > 50).

Generation of an optimized PRM assay panel for SARS-CoV-2 detection – To generate a panel of optimal PRM assays for SARS-CoV-2 protein detection, we tested the 98 established assays in a supernatant sample of SARS-CoV-2 infected Vero E6 cells. In this biological sample, we could detect 57 endogenous virus peptides representing 5 virus proteins (SPIKE, NCAP, VME1, ORF9B, NS8). Because targeting many peptides can challenge the cycle time of the mass spectrometer, particularly when short LC gradients are used, the list of 57 peptides was further prioritized. For proteins with more than 5 detected peptides, we selected the ones that provide the highest endogenous PRM-MS2 signal using the top 6 fragment ions from the experimental spectrum of the spectral library. Further, we gave priority to peptides that were unique for SARS-CoV-2 (for criteria see below; Supplemental Table S3). Finally, we derived a panel of 23 optimal PRM assays for SARS-CoV-2 detection using nano-flow PRM and a 50-minutes linear gradient length (Supplemental Table S3). In the micro-flow PRM measurements, with only 15-minutes linear gradients, we decreased the PRM assay number to 21 peptides and excluded the targeted detection of retention time peptides.

PRM LC-MS/MS measurements

Targeted PRM measurements were performed on fractionated cell line samples, the isotopically labeled synthetic peptide mixture, a supernatant sample and dilutions of SARS-CoV-2 infected Vero E6 cells, and on two clinical cohorts of respiratory specimens using a nano-flow and a micro-flow system. MS-system carry was monitored and minimized throughout all swab sample measurements by performing blank injections between each sample.

Nano-flow – Nano-flow PRM measurements were performed using a 50 min linear gradient as described in the main methods for the spectral library generation but operating the Fusion Lumos in PRM mode. Targeted MS2 spectra were acquired at 60 k resolution within 100-2,000 m/z, after HCD with 30 % NCE, and using an AGC target value of 4e5 charges, a maxIT of 118 ms and an isolation window of 1.3 m/z. The number of targeted precursors was adjusted to a cycle time of at maximum 2 s. In initial evaluation runs using the isotopically labeled synthetic peptide mixture and the supernatant sample, 113 theoretical tryptic SARS-CoV-2 peptides were targeted in different charge states and in light and/or heavy form requiring partition of the precursor list over 6 to 8 injections per sample. For the PRM analysis of the dilution series samples and nasopharyngeal swab samples, only 23 optimal SARS-CoV-2

peptide precursors, plus 11 iRT peptide precursors, were targeted within a single PRM measurement and with a 6 minutes schedule retention time window.

Micro-flow **–** Micro-flow PRM measurements were performed using a 15 min linear gradient as described in the main methods for the spectral library generation but operating the Fusion Lumos in PRM mode. Targeted MS2 spectra were acquired at 60 k resolution within 100-2,000 m/z, after HCD with 32 % NCE, and using an AGC target value of 1e5 charges, a maxIT of 118 ms and an isolation window of 1.3 m/z. The number of targeted precursors was adjusted to a cycle time of at maximum 0.9 s to obtain at least 6-7 data points per peak. For peptides of monkey TMPRSS2 that have not been identified in DDA runs, retention time for scheduling was predicted using Prosit. In total, 21 SARS-CoV-2 peptides or 21/15 human/monkey ACE2 and TMPRSS2 peptides were targeted in 1 min wide transition windows except for peptides for which no experimental retention time was available in which case 2 min wide transition windows were employed. No peptides for retention time calibration were scheduled for fragmentation, but PROCAL peptides were spiked into samples to utilize MS1 chromatogram information. For ACE2 and TMPRSS2 PRM measurements, leftovers from 48 high pH RP fractions (~10%) were pooled consecutively into 8 fractions (i.e. fraction 1-6, 7-12, and so on), and respective PRM methods were applied to all 8 fractions of all 4 cell lines.

Data analysis

Exemplary refinement of the gene annotation of Chlorocebus sabaeus/Vero genome **–** Results of the database search of baseline deep proteomes of Vero E6 cells were filtered for genes that were identified with at least two peptides that uniquely map to the human database and therefore hint at a potential misannotation of homologous *Chlorocebus* genes. These ‘human-only’ peptides were mapped to the 6-frame translation of the Uniprot/RefSeq Green Monkey and a Vero cell line genome (ATCC #JCRB0111) (12). For this, the unmasked DNA sequence of *Chlorocebus* (Assembly ChlSab1.1; GCA_000409795.2) was accessed via Ensembl (ftp://ftp.ensembl.org/pub/release-100/fasta/chlorocebus_sabaeus/dna/, on May 6, 2020), while the Vero DNA sequence assembly was downloaded from the NCBI SRA database (BioProject PRJDB2865; https://sra-download.ncbi.nlm.nih.gov/traces/dra2/DRZ/000003/DRZ003174/VeroPE_SGA_200_k5_a0.7.final.scaffolds.fasta). Six-frame translations of the two genomes were performed using the R package seqinr (version 3.4-5) (13). Manual examination identified BRCA1 and SRP9 as genes where multiple ‘human-only’ peptides mapped to a relatively small region (matching region) of the 6-frame translation. Thus, the amino acid sequences around the matching regions in the *Chlorocebus* and Vero *genome* were extracted for these two genes and compared to the potential human homologous protein using Clustal (14). The predicted gene models of the Vero JCRB0111 cell line was kindly provided by the authors of (12). To further validate the expression of SRP9 and exons missed in the annotated BRCA1 genes in RefSeq (exon 9) and the Vero JCRB0111 gene model (exon 2 and 4-7) in Vero E6 cells, the corresponding deep proteome data was re-searched against the RefSeq *Chlorocebus sabaeus* database complemented with the newly identified SRP9 and BRCA1 sequences from

the Monkey and Vero JCRB0111 cell line genome. To examine the quality of the newly identified peptides from SRP9 and BRCA1 that uniquely map to our new SRP9 or BRCA1 gene models, several of their spectra were compared with the Prosit-predicted spectra (10). Similarity scores were computed based on predicted b- and y-ions only, with the help of Skyline (8) and ProteomicsDB's interactive spectrum viewer (https://www.proteomicsdb.org/isv; Schmidt *et al.*, manuscript in preparation).

Analysis of the virus-dose experiment*:* Hits to the reverse and contaminant database were removed. Reporter ion intensities of multiplexed mock and SARS-CoV-2 treated Vero E6 samples were normalized for mixing errors based on the total sums of peptide intensities in each channel. For calculation of normalization factors, only peptides that were quantified in all 9 channels were considered. To calculate protein iBAQ values for each replicate in all three conditions, each iBAQ value reported by MaxQuant (corresponding to the sum of all 9 channels) was proportionally divided among the 9 channels according to the share of the respective TMT reporter ion intensity in the summed TMT intensity. The Perseus software suite (v.1.6.14.0) was utilized to perform correlation analysis, principal component analysis, two-sided Student's t-tests, clustering, Fisher's exact tests, and functional 2D enrichments (15). Statistical tests were corrected for multiple testing applying a permutation-based or Benjamini-Hochberg FDR calculation at 5 % as indicated. For t-tests, reporter intensities were log transformed to achieve a normal distribution, at least 2 valid quantifications per group were required, and equal variances were assumed for each group. S0 was computed in R (v3.6.0, function samr) for each statistical group comparison separately. Unsupervised clustering was performed based on Euclidean distance, and a distance threshold of 2.3 was specified to extract in total 12 clusters with 6 main clusters containing at least 40 proteins. For annotation of functional categories, UniProt Keywords, Human protein atlas (HPA) protein classes, Reactome pathways, Gene Ontology Biological Processes (GOBP), and CORUM complexes were downloaded for Vero E6 (if available) and human proteins on respective websites. For comparison of our data with a published virus-host response study in Caco-2 (16), supplementary data provided on expression changes 24 h post infection with SARS-CoV-2 at 1 MOI were re-analyzed for significantly changing proteins analogous to our data as described above. Potential interactors of viral proteins were identified based on a published list of 332 confident interactors (17). All annotations were mapped to our dataset based on gene names. Data were visualized using Perseus, R, GraphPad, Tableau, or Cytoscape.

PRM data analysis – Nano-flow and micro-flow PRM data were analyzed using the Skyline-daily (64-bit) software (version 20.1.1.83) (8). For all target peptides, the most intense precursor charge state and the 6 most-intense fragment ions were selected automatically by Skyline using the experimental or Prosit-predicted spectral libraries. The raw PRM data were imported into Skyline and peak integration and transition interferences were reviewed manually. If necessary, integration boundaries were manually adjusted and strongly interfered transitions were removed from the complete dataset but keeping at least 5 transitions per peptide.

ACE2 and TMPRSS2 detection in fractionated cell line proteomes: For targeted ACE2 and TMPRSS2 peptide detection in the fractionated cell line samples, we exported from Skyline

the mass accuracy information (“Average Mass Error PPM”) and correlation of fragment ion intensities between the detected ACE2 or TMPRSS2 peptides measured by PRM and the experimental library spectrum (“Library Dot Product”). A positive peptide detection was defined with a Library Dot Product > 0.85 and an Average Mass Error PPM between 4 and -4 ppm, (Supplemental Table S1 and https://panoramaweb.org/SARS-CoV-2.url). If a peptide was detectable in several fractions of a given cell line, the total peptide intensity was computed by summing peptide intensities over all fractions.

Dilution series experiment: The lower limit of detection was investigated for each targeted SARS-CoV-2 peptide within the dilution series experiment (Supplemental Table S3 and https://panoramaweb.org/SARS-CoV-2.url). A global SARS-CoV-2 response (Supplemental Figure 11B) was determined by summing up PRM-MS2 intensities of all detectable light peptides at a given dilution step. The lowest dilution step of still confident detectability was determined by manual inspection. Three peptides that showed a confounding background signal in the blank and/or low concentration samples in the nano-flow dilution series (LNTDHSSSSDNIALLVQ, GFYAEGSR and FLPFQQFGR) were excluded from the analysis of the nano-flow clinical sample cohort 1.

Clinical swab sample cohorts: To discriminate between positive and negative peptide detection in the clinical swab sample cohorts, three parameters were exported from Skyline: i) mass accuracy (“Average Mass Error PPM”), ii) correlation of fragment ion intensities between the light SARS-CoV-2 peptide measured by PRM and the experimental library spectrum (“Library Dot Product”), iii) correlation of fragment ion intensities between the light (endogenous) and heavy (spike-in) peptide measured by PRM (“DotProductLightToHeavy”). Only clinical samples for which at least one light SARS-CoV-2 peptide fulfilled the following criteria were classified as “positive”: Average Mass Error PPM between 4 and -4 ppm, Library Dot Product > 0.85 and DotProductLightToHeavy > 0.90 (Supplemental Table S4 and https://panoramaweb.org/SARS-CoV-2.url). The total SARS-CoV-2 intensity per clinical sample was computed by summing up all light peptide intensities detected positive in a given clinical sample. MS-system carry-over was monitored and minimized throughout all swab sample measurements by performing blank injections between each sample.

Uniqueness of SARS-CoV-2 peptides: In order not to confuse SARS-CoV-2 with other coronaviruses such as the four human endemic coronaviruses (229E, HKU1, NL63 and OC43), which are often the cause of the common cold, or with proteins of entirely unrelated organisms, we evaluated whether peptides targeted in our PRM assay were unique for SARS-CoV-2 (Supplemental Table S3). All theoretical tryptic SARS-CoV-2 peptides with 6 to 36 amino acids were N-terminally extended by R or K (except for peptides on the protein N-terminus). Additionally, peptide variants carrying Leu instead of Ile and vice versa were included. All resulting peptide sequences were compared to a non-redundant protein database including entries from GenPept, Swissprot, PIR, PDF, PDB, and RefSeq (downloaded on May 3, 2020 from https://ftp.ncbi.nlm.nih.gov/blast/db/FASTA/nr.gz) to identify all peptides that map to any other organisms than SARS-CoV-2.

Supplemental Discussion

Comparison of nano- and micro-flow PRM measurements

For detection of SARS-CoV-2 in clinical samples with highly complex and individually varying background proteomes, we set up PRM assays on nano- and micro-flow LC systems. Initially, 57 peptides mapping to five SARS-CoV-2 proteins were detected in supernatants of infected Vero E6 cells by nano-LC PRMs using 1 h gradients. We were aiming to evaluate the feasibility of increasing the sample throughput and robustness of measurements for the screening of clinical samples by employing micro-flow chromatography. Because targeting many peptides can challenge the cycle time of the mass spectrometer, particularly when short LC gradients are used, the list of 57 peptides was reduced to 21 peptides for micro-flow measurements using 15 min gradients.

To characterize the PRM assays further, we in-gel digested dilutions of Vero E6 supernatant, spiked reference peptides at a constant concentration and analyzed the samples by nano-flow and micro-flow PRM starting with the lowest dilution. As an example, Supplemental Figure S11A shows the detailed PRM results for the SARS-CoV-2 unique peptide VGGNYNYLYR of the SPIKE protein. As we already reported previously (18), the signal of the spiked heavy peptide was on average 5-10 times higher on the nano-flow compared to the micro-flow system illustrating the superior sensitivity of nano-flow measurements. The well-known difference in sensitivity is driven mostly by competition for charges during the ionization process in micro-flow and thus quite dependent on the peptide and the corresponding background. For instance, the intensity of the VGGNYNYLYR heavy peptide was 5-7 times higher on the nano-flow compared to the micro-flow system. The lowest input in which the endogenous peptide could be detected was equivalent to 50 ng total protein or 33 virions on the nano-flow LC-MS/MS system and 100 ng total protein or 66 virions on the micro-flow instrument. The number of infectious particles should be taken with caution because we could only quantify the number of infectious virus/ml (~2x10e6) but not accurately determine the ratio between total virus and infectious virus in the supernatant (estimated to be ~5:1). We note further that the discrepancy in factors of MS intensity (5-7x) determined from heavy peptides spiked in directly before MS measurement and the factors of apparent limit of detection (2x) determined in different dilutions for the endogenous peptide are likely due to two factors. First, samples are derived from in-gel digestion of supernatant dilutions rather than dilutions of the identical peptide samples. Therefore, losses during the sample processing are not expected to be equal (in relative terms) across different dilution steps which hampers the direct comparability with regard to detection limits determined in different dilution steps. Second, the absolute quantity injected from different workflow dilutions was different for nano- and micro-flow measurement, which additionally complicates a comparison of detection limits for both LC systems.

Nevertheless, one can generally state that the micro-flow system is less sensitive. At the same time, however, it showed much higher stability of retention times compared to the nano-flow system (Supplemental Figure S11C). This is particularly important when measuring low abundant peptides in proteomic backgrounds of varying total protein content (as observed in

clinical samples; Supplemental Figure S12A) because overloading the column can lead to drastic shifts of retention times. Indeed, this was observed for the nano-flow system when sample loadings exceeded 500 ng, while no such effect was observed for the micro-flow system even at loadings of 10 µg (Supplemental Figure S11C). The micro-flow system thus enables easier scheduling of PRM assays and supports higher sample loadings than nano-flow PRMs, which is especially beneficial for clinical samples with highly varying background, but comes at the cost of sensitivity. This was also apparent in measurements of clinical samples (Supplemental Figure S12C, D). Depending on which heavy reference peptide was inspected, the sensitivity of the nano-flow system was again 5-10 higher than that of the micro-flow system. The variation of the signal peak areas was quite comparable, but as expected the retention time stability was much better on the micro-flow system.

Supplemental References

1. Hoffmann, M., Kleine-Weber, H., Schroeder, S., *et al.* (2020) SARS-CoV-2 Cell Entry Depends on ACE2 and TMPRSS2 and Is Blocked by a Clinically Proven Protease Inhibitor. *Cell* 181, 271-80.e8
2. Thao, T. T. N., Labroussaa, F., Ebert, N., *et al.* (2020) Rapid reconstruction of SARS-CoV-2 using a synthetic genomics platform. *Nature* 10.1038/s41586-020-2294-9
3. Dagley, L. F., Infusini, G., Larsen, R. H., *et al.* (2019) Universal Solid-Phase Protein Preparation (USP3) for Bottom-up and Top-down Proteomics. *Journal of Proteome Research* 18, 2915-24
4. Hughes, C. S., Moggridge, S., Müller, T., *et al.* (2019) Single-pot, solid-phase-enhanced sample preparation for proteomics experiments. *Nature Protocols* 14, 68-85
5. Zecha, J., Satpathy, S., Kanashova, T., *et al.* (2019) TMT Labeling for the Masses: A Robust and Cost-efficient, In-solution Labeling Approach. *Mol Cell Proteomics* 18, 1468-78
6. Carr, S. A., Abbatiello, S. E., Ackermann, B. L., *et al.* (2014) Targeted Peptide Measurements in Biology and Medicine: Best Practices for Mass Spectrometry-based Assay Development Using a Fit-for-Purpose Approach. *Mol Cell Proteomics* 13, 907-17
7. Abbatiello, S., Ackermann, B. L., Borchers, C., *et al.* (2017) New Guidelines for Publication of Manuscripts Describing Development and Application of Targeted Mass Spectrometry Measurements of Peptides and Proteins. *Mol Cell Proteomics* 16, 327-28
8. MacLean, B., Tomazela, D. M., Shulman, N., *et al.* (2010) Skyline: an open source document editor for creating and analyzing targeted proteomics experiments. *Bioinformatics* 26, 966-68
9. Tyanova, S., Temu, T., and Cox, J. (2016) The MaxQuant computational platform for mass spectrometry-based shotgun proteomics. *Nat Protoc* 11, 2301-19
10. Gessulat, S., Schmidt, T., Zolg, D. P., *et al.* (2019) Prosit: proteome-wide prediction of peptide tandem mass spectra by deep learning. *Nature Methods* 16, 509-18
11. Sharma, V., Eckels, J., Schilling, B., *et al.* (2018) Panorama Public: A Public Repository for Quantitative Data Sets Processed in Skyline. *Mol Cell Proteomics* 17, 1239-44
12. Osada, N., Kohara, A., Yamaji, T., *et al.* (2014) The Genome Landscape of the African Green Monkey Kidney-Derived Vero Cell Line. *DNA Research* 21, 673-83
13. Charif, D., and Lobry, J. R. (2007) SeqinR 1.0-2: A Contributed Package to the R Project for Statistical Computing Devoted to Biological Sequences Retrieval and Analysis. In: Bastolla, U., Porto, M., Roman, H. E., *et al.*, eds. *Structural Approaches to Sequence Evolution: Molecules, Networks, Populations*, pp. 207-32, Springer Berlin Heidelberg, Berlin, Heidelberg
14. Sievers, F., Wilm, A., Dineen, D., *et al.* (2011) Fast, scalable generation of high-quality protein multiple sequence alignments using Clustal Omega. *Molecular systems biology* 7, 539
15. Cox, J., and Mann, M. (2012) 1D and 2D annotation enrichment: a statistical method integrating quantitative proteomics with complementary high-throughput data. *BMC Bioinformatics* 13, S12
16. Bojkova, D., Klann, K., Koch, B., *et al.* (2020) Proteomics of SARS-CoV-2-infected host cells reveals therapy targets. *Nature* 10.1038/s41586-020-2332-7
17. Gordon, D. E., Jang, G. M., Bouhaddou, M., *et al.* (2020) A SARS-CoV-2 protein interaction map reveals targets for drug repurposing. *Nature* 10.1038/s41586-020-2286-9
18. Bian, Y., Zheng, R., Bayer, F. P., *et al.* (2020) Robust, reproducible and quantitative analysis of thousands of proteomes by micro-flow LC-MS/MS. *Nat Commun* 11, 157
